# Rampant Genome-Wide Admixture across the *Heliconius* Radiation

**DOI:** 10.1093/gbe/evab099

**Published:** 2021-05-04

**Authors:** Krzysztof M Kozak, Mathieu Joron, W Owen McMillan, Chris D Jiggins

**Affiliations:** 1 Smithsonian Tropical Research Institute, Panamá, Panamá; 2 Department of Zoology, University of Cambridge, United Kingdom; 3 Centre d’Ecologie Fonctionnelle et Evolutive (CEFE), CNRS, Université de Montpellier, Université Paul Valéry Montpellier 3, EPHE, IRD, France

**Keywords:** admixture, adaptive introgression, radiation, phylogenomics

## Abstract

How frequent is gene flow between species? The pattern of evolution is typically portrayed as a phylogenetic tree, yet gene flow between good species may be an important mechanism in diversification, spreading adaptive traits and leading to a complex pattern of phylogenetic incongruence. This process has thus far been studied mainly among a few closely related species, or in geographically restricted areas such as islands, but not on the scale of a continental radiation. Using a genomic representation of 40 out of 47 species in the genus, we demonstrate that admixture has played a role throughout the evolution of the charismatic Neotropical butterflies *Heliconius*. Modeling of phylogenetic networks based on the exome uncovers up to 13 instances of interspecific gene flow. Admixture is detected among the relatives of *Heliconius erato*, as well as between the ancient lineages leading to modern clades. Interspecific gene flow played a role throughout the evolution of the genus, although the process has been most frequent in the clade of *Heliconius melpomene* and relatives. We identify *Heliconius hecalesia* and relatives as putative hybrids, including new evidence for introgression at the loci controlling the mimetic wing patterns. Models accounting for interspecific gene flow yield a more complete picture of the radiation as a network, which will improve our ability to study trait evolution in a realistic comparative framework.

**Significance**

Gene flow and adaptive introgression have been documented across the tree of life, but the overall importance of these processes for the diversity of life remains unclear. We find that admixture between species has been common during the evolution of *Heliconius* butterflies, and was the mechanism spreading multiple advantageous genes. Therefore, we point to gene flow between species as a key force shaping an adaptive radiation.

## Introduction

Interspecific hybridization and the resulting gene flow across porous species barriers are increasingly recognized as major processes in evolution, detectable across the tree of life (Hilario and Gogarten 1993; Feliner et al. 2017). Interspecific gene flow has since been demonstrated in several animal taxa, both at deep (Chen et al. 2017) and shallow temporal scales (e.g., Fontaine et al. 2015). Although hybridization might often result in the production of deleterious combinations of alleles at different loci, introgression can also enable adaptation by providing novel variation that may be favored by natural selection, as demonstrated in the iconic adaptive radiation of Darwin’s finches (Lamichhaney et al. 2015) and in our own lineage (Huerta-Sánchez et al. 2014).

The task ahead is to systematically evaluate the prevalence and importance of interspecific gene flow in fueling speciation in adaptive radiations (Schumer et al. 2014; Feliner et al. 2017). Unfortunately, modeling gene flow requires extensive data and poses greater challenges to computational methods than other processes leading to incongruent signals, such as incomplete lineage sorting or gene duplication (Degnan 2018; Wen et al. 2018). For instance, two studies of the swordtail fishes applied different sequencing and analytical strategies, ultimately reaching dissimilar conclusions on the prevalence of hybridization in *Xiphophorus* (Cui et al. 2013; Kang et al. 2013). The challenge of characterizing introgression in adaptive radiations remains open and requires both taxonomic completeness and sophisticated methodological approaches.

The Neotropical *Heliconius* butterflies present an excellent opportunity to study the incidence and importance of gene flow in a recent adaptive radiation, due to the natural propensity of *Heliconius* and the sister genus *Eueides* to produce hybrids in the wild (Dasmahapatra et al. 2007; Mallet et al. 2007). The loci responsible for their aposematic wing patterns are especially likely to be shared between species, providing a source of genetic variation in a strongly selected trait and thus likely facilitating speciation (Heliconius Genome Consortium 2012; Pardo-Diaz et al. 2012; Enciso-Romero et al. 2017; Jay et al. 2018; Edelman et al. 2019; Massardo et al. 2020) (summarized in supplementary table 1, Supplementary Material online). In the so called *melpomene/cydno/*silvaniform clade (MCS), species in sympatry can share variation at up to 40% of the genome (Kronforst et al. 2013; Martin et al. 2013).

It remains unknown whether hybridization and introgression documented in the relatively young MCS clade (4.5–3.5 Ma) (Kozak et al. 2015) are a universal characteristic of this genus. Recent studies of the genus *Heliconius* based on de novo assembly of genomes (Edelman et al. 2019 Seixas et al., 2021 ) and transcriptomes (Zhang et al. 2019) have identified instances of admixture in other groups, but looked at single individuals in less than half of the 47 recognized species. Furthermore, a study of *Heliconiushermathena* revealed that a phenotype suggestive of introgression is in fact determined by an ancestral allele expressed in multiple species (Massardo et al. 2020). Full understanding of the frequency of interspecific admixture events requires comprehensive analysis across the diverse species and phenotypic races in the genus.

**Fig. 1.**
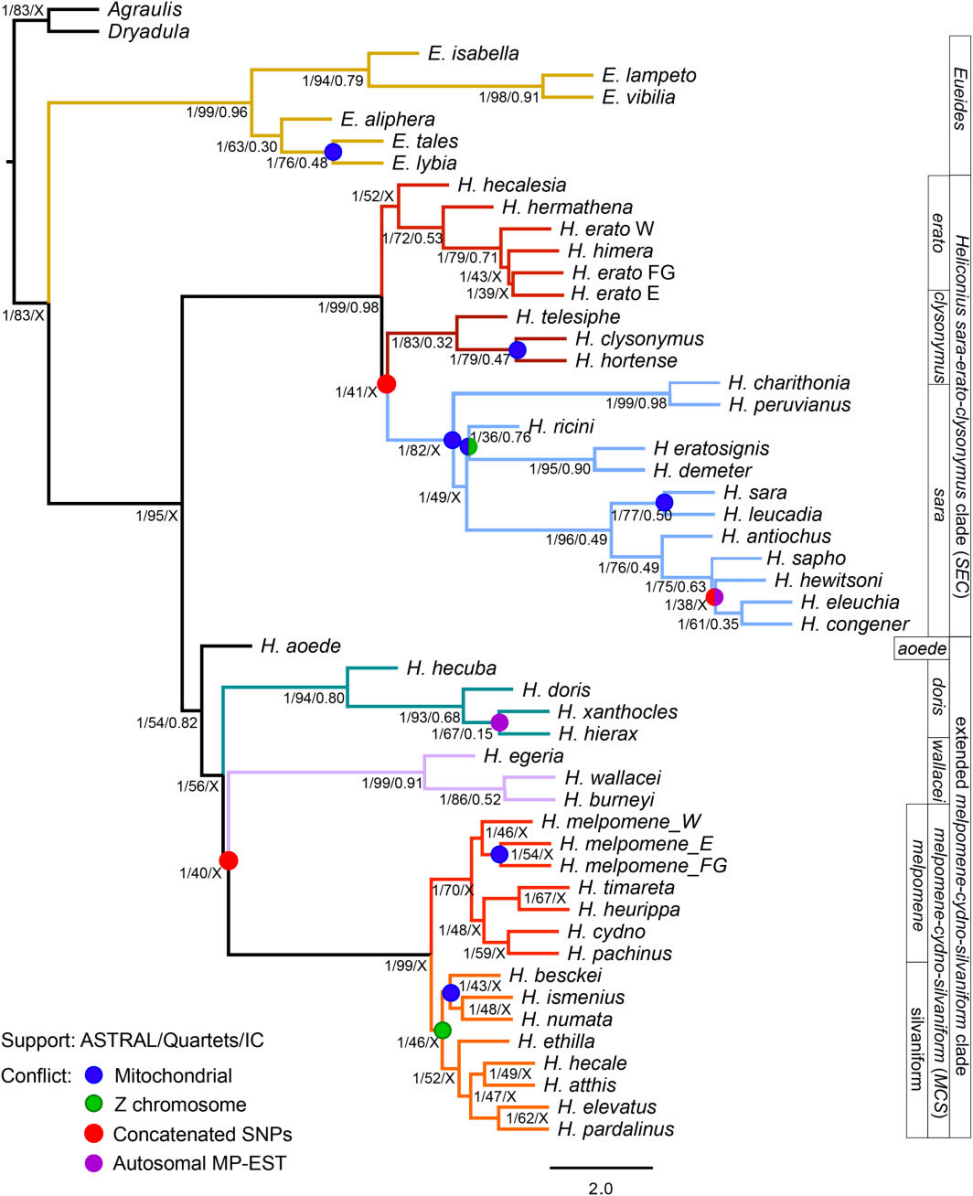
Bifurcating model of Heliconiini phylogeny obscures the underlying incongruence. The topology was inferred by the multispecies coalescent method ASTRAL-III from 6,725 orthologous autosomal genes. All nodes, except for the *CHT* clade are supported in 100% bootstrap replicates. In addition, three types of support values are presented: ASTRAL branch support for a quadripartition (on a 0–1 scale); percentage of quartets in individual gene trees containing a specific node; and the Internode Certainty measure of gene tree incongruence (0–1 scale; X indicates the node is not found in the consensus). Colored circles indicate conflict between the topology of the ASTRAL autosomal tree, and the estimates from: mitochondrial data under ML (blue); sex-linked genes (Z chromosome) in ASTRAL (green); concatenated SNPs under ML (red); the alternative coalescent method MP-EST (violet). Branch lengths are in coalescent units, and arbitrarily set to 1.0 for the terminal branches. Branch colors correspond to previously defined clades (Kozak et al. 2015): red—*Heliconius melpomene*/cydno; orange—silvaniforms (MCS); violet—*Heliconius wallacei*; green—*Heliconius doris*; blue—*Heliconius sara*; crimson—*Heliconius clysonymus*; scarlet—*Heliconius erato*; brown—*Eueides*.

Here, we generate a comprehensive whole-genome resequencing data set of 145 individuals of 40 among the 47 recognized *Heliconius* species, and six out of 12 *Eueides*, encompassing nearly an entire radiation at a continental scale (fig. 1 and supplementary file 1, Supplementary Material online). With this expanded data set, we investigate the prevalence of hybridization, attempt to quantify its extent across the radiation, and compare the processes producing discordance. We demonstrate varied amounts of phylogenetic incongruence (i.e., conflict between gene trees [Degnan 2018] related to heterogenous levels of gene flow among species). We show that a misleadingly well-supported and resolved tree can be recovered despite incongruence, and that previously unknown, complex hybridization events can thus be missed. Although instances of hybridization across the genome, particularly at adaptive loci, are found across the radiation, we demonstrate that they are far more frequent among the relatives of *Heliconiusmelpomene.*

## Results

### Genomic Trees for the Heliconiines

We first constructed a bifurcating autosomal phylogeny. Mapping genome-wide short read data from 145 individuals in 48 species (supplementary file 1 and table 3, Supplementary Material online) to the *H. melpomene* reference allowed us to recover 6,848 autosomal and 416 sex-linked high quality, orthologous CDS alignments, respectively (of which 6,725 and 406 include at least one outgroup sequence). The mean length of a quality-trimmed autosomal alignment was 1,387 base pairs (supplementary table 4, Supplementary Material online), and the average parametric aLRT support for the estimated maximum likelihood gene trees was 0.675 ± 0.060 with all samples included. Filtering for autosomal exome sites with biallelic, nonsingleton SNPs without missing data produced a 122,913 bp supermatrix. This underpins a maximum likelihood tree resolved with full bootstrap support, except for uncertain placement of the *Heliconiusclysonymus/hortense/telesiphe* clade (bootstrap support 62/100), which is also placed differently in the coalescent analyses (fig. 1 and supplementary fig. 1, Supplementary Material online). ASTRAL-III and MP-EST, which infer the species tree from gene trees, also yield highly resolved and supported phylogenies (fig. 1 and supplementary fig. 2, Supplementary Material online). The multispecies coalescent (MSC) trees differ from each other at only two relatively recent splits out of 56. The concatenation phylogeny differs from the ASTRAL at three nodes, and from MP-EST at two (fig. 1).

### Genome-Wide Incongruence and Discordance

Although the species tree topologies recovered by various approaches from the autosomal markers are very similar, multiple indices show incongruence among individual gene trees. The Robinson–Foulds pairwise distance is high: 0.745 out of 1.0 for the autosomal phylogenies and 0.699 for the sex-linked loci, indicating that any two gene trees are very likely to contain multiple differing nodes. Among the 56 nodes separating species and major subspecies, less than a half (26) are resolved in an autosomal majority rule consensus tree (supplementary fig. 4, Supplementary Material online). The relative tree certainty (Salichos et al. 2014) on a 0–1 scale is a low 0.322 when using all gene trees, and increases to 0.397 for the 1,000 best gene trees. Many branches with high support in the coalescent trees score low on the internode certainty (IC) measures (IC/ICA; supplementary fig. 4, Supplementary Material online). Brower and Garzón-Orduña (2018) suggested that incongruence reported previously (Kozak et al. 2015) was an artifact of missing data. Here, we present nucleotide matrices that are nearly complete (>96%). Although modern statistical phylogenetic methods are typically robust to far higher levels of missing data (Wiens and Morrill 2011; Roure et al. 2013), we still find substantial discordance and incongruence, which shows they are not merely artifacts.

Conflicts are further highlighted by the varied quartet support, whereby many of the nodes reported as certain by ASTRAL are only found in a fraction of the gene trees (second set of support values in fig. 1). This discrepancy is especially exacerbated in the low quartet support for the position of species in the *MCS* clade, as well as at the placement of the small clades of *Heliconiusaoede*, *Heliconiuswallacei*, *Heliconiusdoris*, and *H.clysonymus*. The statistics are not strongly affected by the exact choice of markers. When the 6,367 single exons are used instead of entire genes, the total normalized quartet score for the entire ASTRAL tree decreases only slightly from 0.847 to 0.806, while the quartet support of individual nodes changes by no more than 10 percentage points (supplementary fig. 3, Supplementary Material online).

We found that the Z chromosome gene trees are more congruent with each other (supplementary figs. 5 and 6, Supplementary Material online) and more concordant with the coalescent species trees (supplementary figs. 7 and 8, Supplementary Material online), than are the autosomal trees (Wilcoxon's test, *P *=* *3×10^−11^). Notably, many nodes within the *MCS* clade are resolved among the Z chromosome trees, and the *H. melpomene/cydno* group is monophyletic (supplementary fig. 4, Supplementary Material online), unlike in the consensus of autosomal gene tree topologies, where it is mixed with silvaniform relatives (supplementary fig. 4, Supplementary Material online). Similarly, the whole mitochondrial phylogeny was both well-supported, and conflicted with the MSC trees at different levels of divergence (fig. 1 and supplementary fig. 9, Supplementary Material online).

### Hybridization between Species Has Been Common throughout the Radiation of *Heliconius*

**Fig. 2.**
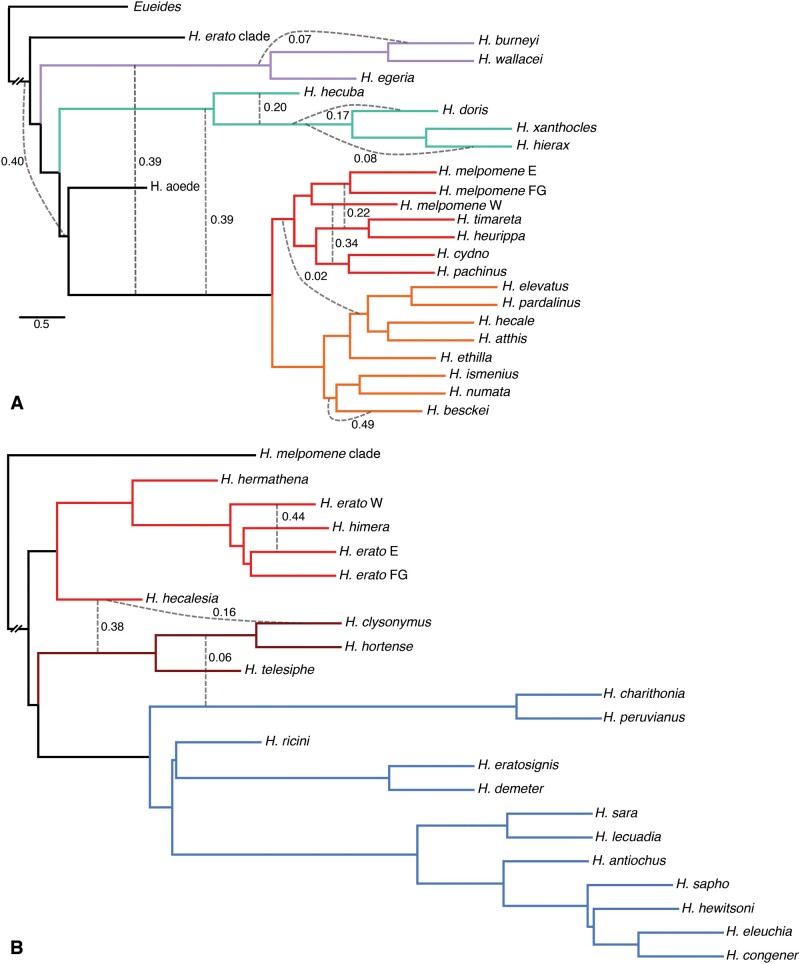
Evidence of introgression is found across the entire *Heliconius* radiation. Networks inferred under maximum pseudolikelihood (MPL) based on 6,725 autosomal ML gene trees distinguish between introgression and incomplete lineage sorting, revealing several admixture events. Numbers on the edges indicate the inheritance probabilities (Wen et al. 2018), which correspond to the proportion of the data supporting the grouping. (*A*) The analysis of *Heliconius melpomene* and cognates reveals previously undetected introgressions closer to the root of the tree. Known events in the MCS clade are recapitulated, demonstrating sensitivity of the approach. (*B*) Fewer admixtures occurred in the evolution of the *Heliconius erato/sara* clade, but *Heliconius hecalesia* may be a recent hybrid.

**Fig. 3.**
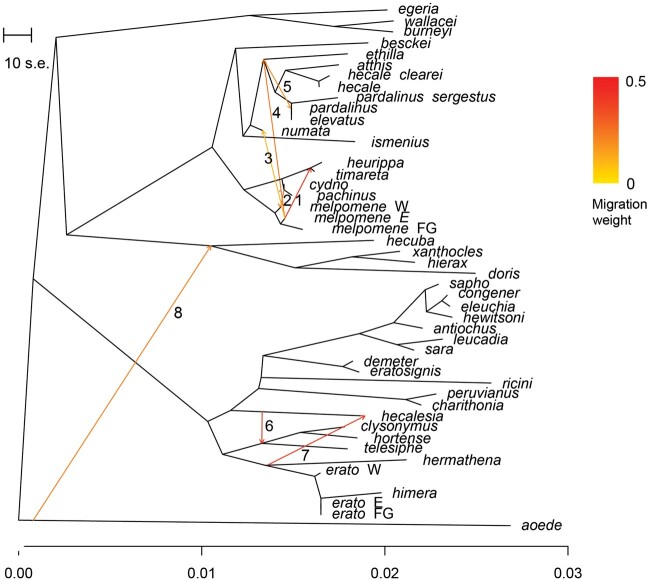
The extent of interspecific gene flow varies across the tree. TreeMix inference of splits and mixture from autosomal SNPs. Migration edges (1–8) are inferred on a phylogenetic tree built from allele frequencies under a Gaussian genetic drift approximation. Colors of the edges correspond to the proportion of the genome exchanged.

For the first time, we test for gene flow across the entire radiation, including nearly all species (*N* = 27) in clades other than the previously studied *MCS* (*N* = 13). Across the radiation, an inference of species networks reveals a pattern of gene sharing within all major clades of the radiation (figs. 2 and 3 and supplementary file 3, Supplementary Material online). Both the coalescent network (PhyloNet, fig. 2*A*) and the admixture graph (AG) (TreeMix, fig. 3) support the known gene flow between East Andean races of *H. melpomene* and *Heliconiusheurippa/Heliconiustimareta* (figs. 2 and 3, edge 1), as well as between the Western races of *H. melpomene* and *Heliconiuscydno*/*Heliconiuspachinus* (fig. 3, edge 2). The inferred admixture edges show gene flow at a substantial proportion of the surveyed loci (corresponding to the inheritance probability *γ*; Zhang et al. 2019): 0.34 for *H. melpomene/H. cydno* and 0.22 *H. melpomene–H. timareta*. Both methods show gene flow between silvaniforms and the *H. melpomene/cydno* clade, although compared with the MPL network, the AG suggests more events at a larger proportion of the loci (fig. 3, edges 3–5). Conversely, the network contains more admixture events in the deeper past.

Ample evidence is found for gene flow among other clades and in the deeper past. Gene flow with inheritance probabilities of 0.39 is inferred between *MCS*, and both clades of *H. wallacei* and *H. doris* (fig. 2*A*), along extensive gene flow within the latter two clades. The two inference methods suggest large disagreement on the placement of *H. aoede* (*γ* = 0.40, fig. 2*A*), but suggest gene flow either from *H. aoede* to the *Heliconiushecuba* clade (AG), or a “ghost lineage” linked to a clade of *H. aoede* and *MCS* (MPL). Finally, the most likely network estimated for the genus *Eueides* contains three reticulations (supplementary fig. 10, Supplementary Material online), all of which connect to a “ghost taxon” (Wen et al. 2018).

### Mosaic Genomes in the Heliconius erato Clade

**Fig. 4.**
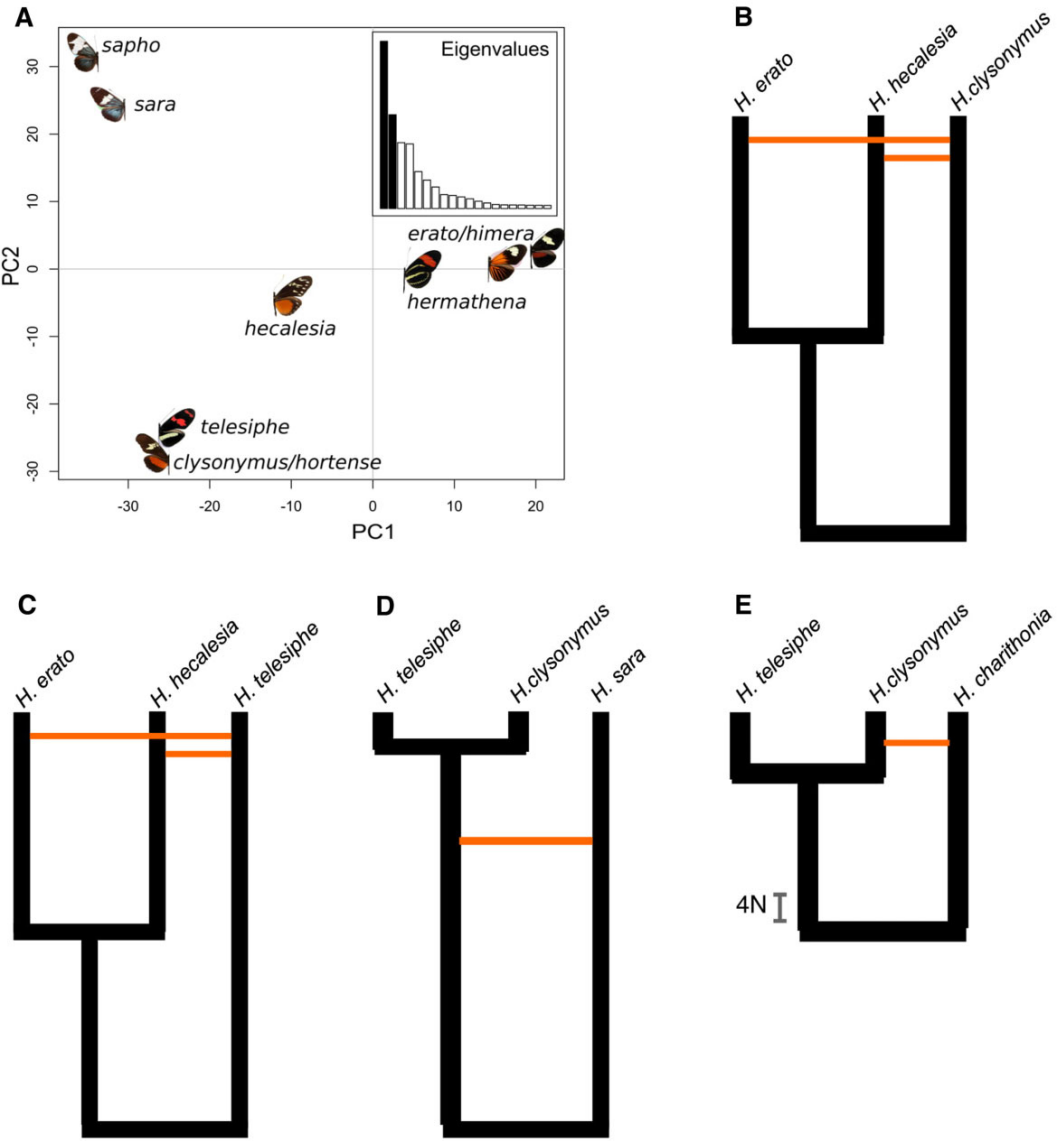
Ambiguous genomic composition of *Heliconius hecalesia*. (*A*) Although usually recovered as the sister species of *Heliconius hermathena* and *Heliconius erato* in bifurcating phylogenies, *H. hecalesia* shares variation with the clades of *Heliconius sara* and *Heliconius clysonymus.* Principal Component Analysis of variation in the autosomal SNPs within the *H. erato/sapho* clade. First two PCs account for over half of the variation. (*B*) Models of divergence history in the *H. erato/Heliconius telesiphe* group inferred by PHRAPL, where orange edges indicate gene flow. Models including up to five terms for coalescence, gene flow and variation in population sizes were fitted to four sets of three taxa, extracted from the 6725 gene trees. The bar in panel 4E shows time in units of 4N (diploid population size; see supplementary table S6, Supplementary Material online).

Proportionally fewer admixture events are identified in the network of the *SEC* clade (three interspecific admixture edges among 21 lineages; fig. 2*B*) than among other *Heliconius* (10 edges between 23 lineages). Nonetheless, support for admixture in this lineage is strong as well. In particular, *Heliconiushecalesia* shares a large portion of its genome with either the ancestor of the (H. telesiphe,(hortense, clysonymus)) clade (*γ* = 0.38, fig. 2*B*), or just with *H. clysonymus* (*γ* = 0.16). Furthermore, there is some evidence for an exchange between the *CHT* and *Heliconiussara* clades (*γ* = 0.06). The TreeMix AG also uncovers the *CHT*-*H. hecalesia* admixture, but places both in different positions in the tree, such that *H. hecalesia* appears more closely related to the *H. sara* clade, and admixes with both *CHT* and *H. erato* (fig. 3, edges 6 and 7). Although grouped with *H. erato* in simple trees (figs. 1–3), *H. hecalesia* appears to be nearly equally diverged from the clades of *H. erato* and *H. clysonymus* (fig. 4*A*), and the support for the placement of *H. hecalesia* with either lineage is nearly equivocal among individual gene trees (quartet score 52/100). Similarly, the position of the* CHT* triplet, often placed with *H. hecalesia*, is the only unsupported branch in the autosomal ML phylogeny (fig. 1 and supplementary fig. 1, Supplementary Material online). No definite placement of *H. hecalesia* and the *CHT* clade is found in a majority of gene trees, as evidenced by consensus and DensiTree plots (IC = 0; supplementary figs. 4 and 11, Supplementary Material online).

The admixture during in the evolution of *H. hecalesia* and the *CHT* clade is evident in the pattern of variation among rooted triplets of taxa, examined using the *D* statistic (Durand et al. 2011) (table 1). The results are highly positive and statistically significant for all tests where *H. hecalesia* is the recipient of admixture from either the *H. clysonymus* or *H. sara* clades (table 1). However, consistent with the phylogenetic patterns, there is evidence for stronger gene flow between *H. hecalesia* and *H. clysonymus* (*D *=* *0.35; *P *<* *0.0001) or *H. hortense* (*D *=* *0.38; *P *<* *0.0001) than the very differently patterned *H. sara* (*D *=* *0.17; *P *<* *0.0001).

**Table 1:** *D*-Statistic Values (ABBA/BABA Tests) of Admixture between *Heliconius hecalesia* and Relatives

*P_1_*	*P_2_*	*P_3_*	*D*	Error(*D*)	*P* value
*erato* FG	*hecalesia*	*clysonymus*	0.349	0.009	<0.0001
*erato* FG	*hecalesia*	*hortense*	0.383	0.009	<0.0001
*erato* FG	*hecalesia*	*telesiphe*	0.269	0.008	<0.0001
*erato* FG	*hecalesia*	*charithonia+peruvianus*	0.208	0.005	<0.0001
*erato* FG	*hecalesia*	*sara+leucadia*	0.170	0.005	<0.0001
*erato* East	*hecalesia*	*clysonymus*	0.354	0.009	<0.0001
*erato* East	*hecalesia*	*sara+leucadia*	0.178	0.004	<0.0001

Note.—*P*_2_ and *P*_3_ are the taxa hypothesized to exchange variants, while the outgroup is always *Heliconius melpomene*. Positive *D* values are evidence for admixture after accounting for ILS. The tests are performed on autosomal SNPs and *P*-values are calculated by block jacknifing.

Explicit coalescent modeling also favors models where admixture occurs across species boundaries (fig. 4*B*–*E*). In general, these models are consistent with the network and the AG. Specifically, the *CHT* clade is at the nexus of admixture events, exchanging alleles with *H. erato* and *H. hecalesia* (fig. 4*B* and *C*); *H. sara* (fig. 4*D*); and *Heliconiuscharithonia* (fig. 4*E*). For each triplet, one model with gene flow was strongly preferred (wAIC = 1), although the inferred rate of gene flow between lineages is a low value of 0.1 4*Nm*, not showing any of the variation in the amount of admixture reflected by the network *γ* values. The estimates of divergence times are inconsistent between models, as the inferred time of coalescence between *CHT* and the sister clade of *H. sara* varies with the exact choice of species (fig. 4*D* vs. *E*).

### Adaptive Introgression at the Wing Pattern Loci

**Fig. 5.**
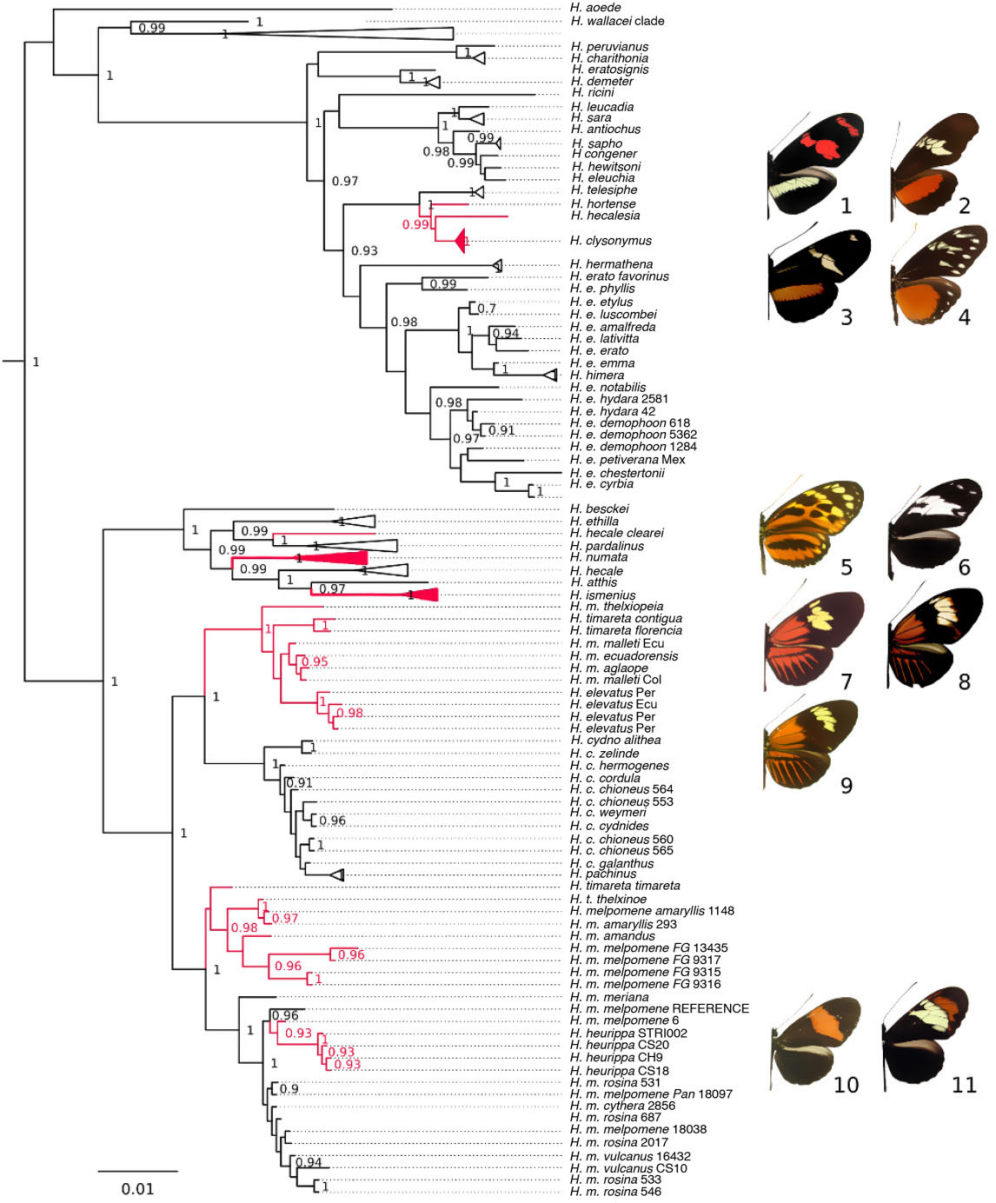
Discordance at the red patterning locus. Branches in positions varying from the species tree are labeled red. The ML tree was estimated for a 20 kb region on the *optix* scaffold (HE670865:360,000:380,000), containing the intervals most strongly associated with wing patterns in *Heliconius erato* and *Heliconius melpomene*. Intraspecific relations for species not discussed in text are collapsed. Outgroups and parametric support values <0.9 not shown. 1. *Heliconius telesiphe sotericus*, 2. *Heliconius hortense*, 3. *Heliconius clysonymus hygiana*, 4. *Heliconius hecalesia formosus*; 5. *Heliconius hecale felix*, 6. *Heliconius hecale clearei*; 7. *Heliconius timareta timareta*, 8. *Heliconius melpomene malleti*, 9. *Heliconius elevatus*; 10. *Heliconius melpomene melpomene*, 11. *Heliconius heurippa*.

Our nearly exhaustive sampling of *Heliconius* species provides a uniform framework in which to gauge the amount of introgression across the functionally important loci that modulate adaptive phenotypic variation. Topologies around wing pattern loci differ from the species tree (*P *<* *0.001, SH test) and in many cases, primarily at the *optix* and *cortex* loci, show multiple departures (supplementary table 5, Supplementary Material online). The majority of the differences are found in the *MCS* clade, where introgression reaches considerable complexity across genomic and geographic regions (Wallbank et al. 2016; Enciso-Romero et al. 2017). Among *Eueides* and in the small clades of *H. aoede*, *H. doris*, and *H. wallacei*, there is no discordance observed at the pattern loci (e.g., fig. 5), despite evidence for gene flow in other parts of the genome (figs. 2 and 3).

The clustering of *H. hecalesia* and *H. clysonymus/H. hortense* is the only case where there is strong evidence for adaptive introgression among the 19 species of the *SEC* clade*.* Sequences from the three species indeed cluster exactly at the 360,000:380,000 interval of scaffold HE670865 (aLRT > 0.95; *P *<* *0.001, SH test), which aligns to the specific region of the *optix* locus controlling red patterns in *H. erato* (Supple et al. 2013) (fig. 5). This indicates that the alleles governing the red pattern in the three species are more similar than expected from the autosomal phylogenies. *H. hecalesia/clysonymus/hortense* cluster also at the *wntA* interval (HE667780: 450,000:490,000) (Martin et al. 2012), to the exclusion of the phenotypically more different *H. telesiphe*.

Considering the heterogeneity observed at the rest of the genome, the discordance in the regions associated with wing patterning may be a product of ILS and not hybridization. We tested this possibility by comparing Bayesian species tree and species network models. At an interval within the *optix* locus (i.e., red patterns; table 2), we find strong support for the network model over the simple tree model (Bayes Factor = 242), and 99.8% of the posterior estimates are networks with at least one admixture edge (*µ *=* *3.92, *σ *=* *1.47). However, the posterior includes 480 topologies, the most common found only in 11.64% of the posterior. This topology (supplementary fig. 12, Supplementary Material online) implies six incidences of gene flow throughout the *SEC* clade, and places *H. hecalesia* in a soft polytomy in the *CHT* clade. In addition, the inferred age of the *CHT* clade (1.6–1.4 Ma; supplementary fig. 12, Supplementary Material online) is much lower than expected from a relaxed molecular clock estimate (4.5–2.7 Ma) (Kozak et al. 2015). Although this discrepancy could be caused by the use of a strict clock here, all the other split times are consistent between the strict and relaxed clock estimates.

At the *Cr* interval (*cortex*: yellow patterns), there is similarly overwhelming support for a network structure over a tree (BF = 411), and 99.4% of the posterior are networks with an average of 3.24 reticulations (*σ*=1.54). Unexpectedly, the most frequent topology (7.86%; supplementary fig. 13, Supplementary Material online) does not place *H. charithonia* with *H. sara*, and contains a single admixture from the ancestor of the *CHT* clade. At *wntA* (the shape locus) the preferred model is also a network (Bayes Factor = 28), and 99.1% of all posterior estimates contain admixture edges (*µ* = 2.58, *σ* = 1.98). Among the most probable networks in the Bayesian posterior, *H. hecalesia* is placed with the *CHT* clade, and 75% of the networks imply gene flow between this clade and *H. charithonia* (supplementary fig. 14, Supplementary Material online).

**Table 2:** Major Wing Pattern and Color Loci in *Heliconius*

*Heliconius Melpomene*	*Heliconius erato*	*Other*	Genes	Scaffold	Phenotype	Key References
*B*	*D*	*Br/G* ^a^	*optix*, putative enhancers	HE670865	Red on HW and FW, ventral brown patterns	Reed et al. (2011), Supple et al. (2013), Van Belleghem et al. (2017), and Wallbank et al. (2016)
*Yb/Sb/N*	*Cr*	*P* ^b^	*cortex*, putative enhancers, and possibly nearby genes.	HE667780	Yellow/white on HW and FW	Joron et al. (2006), Nadeau et al. (2016), Enciso-Romero et al. (2017), and Van Belleghem et al. (2017)
*Ac*	*Sd*		*WntA*, putative enhancers	HE668478 HE669520	Pattern shape	Martin et al. (2012) and Mazo-Vargas et al. (2017)
*Ro*	*Ro*		possibly *vvl* or *rsp3*	HE671554	FW band shape	Morris et al. (2019) and Van Belleghem et al. (2017)
*K*	*K*		*aristaless2*	HE671246 HE670889	White/yellow switch	Westerman et al. (2018)

Note.—Color pattern loci are historically named differently in various species (Sheppard et al. 1985). However, more recent research has demonstrated that loci that have been defined from intraspecific crosses in different species map to homologous regions of the genome (e.g., see Joron et al. [2006]). Moreover, candidate protein-coding genes have been identified and, in some cases, the intervals containing functional variation have been localized (see Key References). Scaffold numbers refer to the *Hmel v1* assembly. HW, hindwing; FW, forewing.

a Brown patterns in *Heliconius cydno* and *Heliconius pachinus* (Chamberlain et al. 2011).

b The *Pushmipullyu* supergene controlling most of the wing patterning in *Heliconius numata* (Joron et al. 2011).

In the *MCS* clade, in addition to corroborating previous reports of introgression around color pattern regions, we identify several new cases. For instance, at the *cortex* locus (see table 2), which is responsible for the diverse white and yellow patterns across the genus (Nadeau et al. 2016), *H. melpomene* and *H. timareta* alleles cluster with silvaniforms (scaffold HE667780:310,000:330,000; aLRT > 0.95). At the *wntA* locus (HE668478:450,000:490,000), sequences of *H. heurippa* cluster with *H. cydno*, upholding the view that speciation of the former involved a yellow-patterned race of *H. cydno* (Enciso-Romero et al. 2017), although the rest of the data places *H. heurippa* unequivocally as sister to *H. timareta* (figs. 1–3). Most of the variation in the *optix* region is consistent with the genome-wide lack of resolution in the *H. melpomene/cydno*/silvaniform clade and confirms known events. The greatest number of discordant branches are among the *H. melpomene/cydno* clade at 360,000:380,000 (fig. 5), the section controlling both *H. melpomene* (Wallbank et al. 2016) and *H. erato* red ray patterns (Supple et al. 2013; Van Belleghem et al. 2017). Intriguingly, alleles from *H. hecale clearei* cluster with the *Heliconiuspardalinus/Heliconiuselevatus* sequences in eight out of 60 windows on the *optix* scaffold, perhaps related to the complete loss of orange patterning in this uniquely black and white silvaniform (fig. 5).

## Discussion

### In-Depth Sampling Reveals Widespread Admixture

We interrogated an extensive data set of 6,725 autosomal genes sequenced in nearly all species of a continental-scale adaptive radiation to investigate the prevalence of genome-wide admixture. We identified up to 13 cases of gene flow between species as a major source of phylogenetic incongruence (fig. 2), and demonstrated that admixture shaped the evolution of *Heliconius* throughout their history. Coalescent modeling revealed admixture between deeply diverged lineages, as well as a complex history of gene flow in the *SEC* clade of the genus. Although *Heliconius* is recognized as a foremost example of interspecific gene flow, most of the studies (reviewed in supplementary table 1, Supplementary Material online) focused on *H.melpomene* and relatives, known to hybridize in the wild with notable frequency (Mallet et al. 2007). Recent studies highlighted new cases in other clades within the genus (Edelman et al. 2019; Zhang et al. 2019), but limited taxonomic and geographic representation of *Heliconius* diversity made it difficult to assess reliably how many species have admixed (Thawornwattana et al. 2021). Here, we include 40/47 species and highlight the importance of admixture in shaping this complex radiation across time. We used the previously investigated clade of *H. melpomene, H. cydno* and silvaniforms as a test case, where our approach supports other work documenting extensive admixture, including: hybridization during speciation of *H.heurippa* (Salazar et al. 2008, 2010); admixture between *H. cydno/timareta* and subspecies of *H. melpomene* (Martin et al. 2013; Nadeau et al. 2013; Enciso-Romero et al. 2017); the exchange between *H. melpomene*, *H. ethilla* group of silvaniforms, and ultimately *H. elevatus* (*Heliconius* Genome Consortium 2012; Wallbank et al. 2016). The fact that we can detect known events increases our confidence in the detection of additional instances across the radiation.

Inclusion of all species in the *SEC* clade made it possible to pinpoint the extensive admixture between *H. hecalesia* and 1) the ancestor of the *H. clysonymus* clade (*γ* = 0.38); 2) *H. clysonymus* itself (*γ* = 0.16). Adaptive gene flow between the three species is plausible, as *H. hecalesia* is sympatric with the other two species in parts of its range (Rosser et al. 2012). *H. clysonymus* × *H. hecalesia* and *H. hortense* × *H. hecalesia* hybrids have been found in the wild (Mallet et al. 2007). To a lesser extent, some degree of gene flow is certain between *H. clysonymus* and *H. sara* clades, although even with rich data it remains difficult to reconstruct specific events when several recently diverged species are involved, as the exact parameter values in the coalescent models depend on the sampling of taxa (fig. 4*D* and *E*). The problem is especially acute in the reconstruction of introgression histories at the wing pattern loci, where no specific topology is strongly supported, and even top-scoring networks are difficult to interpret given the differences in wing phenotypes of putatively introgressing species (fig. 5:1–4 and supplementary figs. 12–14, Supplementary Material online). As variation in *Heliconius* wing patterns appears to be governed by short regulatory elements that differ even between mimics (Concha et al. 2019), detailed investigation will be necessary to identify the specific functional regions within the broader intervals investigated here. Nonetheless, *Heliconius* are unusual in that introgression of unlinked loci enables rapid evolution of complex patterns, which comprise a patchwork of elements sometimes derived from different sources (Wallbank et al. 2016). Many genomic studies of interspecific gene flow have found introgressions of small genome regions driven by natural selection on beneficial alleles, such as multiple abiotic tolerance factors in *Helianthus debilis* into *Helianthusannuus* (Whitney et al. 2010), the hypoxia resistance *EPAS1* haplotype (Denisovans → anatomically modern Tibetans) (Huerta-Sánchez et al. 2014), the *ALX1* alleles determining diverse beak shapes among Darwin's finches (*Geospiza*) (Lamichhaney et al. 2015), or the *Agouti* variant conferring protective coat color (*Lepus americanus* → *Lepustimidus*) (Giska 2019). Only in a few other systems is there evidence for adaptive introgression at multiple loci, including hominins (reviewed by Gokcumen 2020), and the *Lonchura* finches (Stryjewski and Sorenson 2017). Similar to *Lonchura*, the evolution of the key adaptive trait in *Heliconius* (patterning) has involved introgressions at multiple loci and between different combinations of species.

### Challenges of Inferring Interspecific Gene Flow

While admixture is rampant, it remains difficult to describe it with precision. Even though all approaches suggest that gene flow occurred, the exact sources and direction are not estimated consistently between methods. The PhyloNet maximum pseudolikelihood (MPL) networks contain 13 reticulation edges (fig. 2), five of which are also recovered by the TreeMix AG (fig. 3): *H. hecalesia—CHT* clade; *H. hecalesia—H. erato*; *H. melpomene—H. timareta*; *H. melpomene—H. cydno*; *H. melpomene* clade—silvaniforms. The TreeMix AG does not detect the exchanges in and between the small *H. egeria* and *H. hecuba* clades, or some of the events previously documented between species of the silvaniform group and *H. melpomene* (Jay et al. 2018; Zhang et al. 2016). The discrepancies are expected between two widely different techniques, as the TreeMix AG algorithm assumes that the underlying sequence of events was largely tree-like (Pickrell and Pritchard 2012). The AG approach, based on allele frequencies, was designed with assumptions more appropriate at the level of recently diverged taxa and may be affected by issues of multiple substitution. Similarly, the presented *D* statistics need to be taken with caution. Although the factors affecting the specificity of *D* have not been formally determined, it is likely to be affected in clades more distant from the *H. melpomene* reference genome, as worse read mapping results in lower overall number of sites for comparison (supplementary table 3, Supplementary Material online) and thus possibly an unfavorable signal-to-noise ratio. In comparison, the sensitivity of the *D* statistic decreases both when the population size is large relative to the divergence time, as is the case for many widespread *Heliconius* species, and when gene flow was ancient (Zheng and Janke 2018). More surprising is the disparity between two approaches computing over gene trees, MPL networks and PHRAPL. In case of the latter, the ability to evaluate a large number of models with an extensive data set of thousands of gene trees comes at the cost of less accurate parameter estimates, e.g. when compared with Approximate Bayesian Computation approaches (Jackson et al. 2017). Furthermore, the computational burden is reduced by limiting the questions to rooted triplets of taxa and subsampling intraspecific allelic diversity, thus losing many of the benefits of comprehensive sampling.

Other recent studies of introgression among *Heliconius* encountered similar difficulties. For instance, an analysis of whole genomes of 20 species (Edelman et al. 2019) identified the same key patterns (e.g., uncertain placement of *H. hecalesia*; gene flow *H. hecalesia—CHT*; *H. melpomene—*silvaniforms), but with overall low confidence and without the ability to ascertain if the proposed events involved unobserved lineages. Recent application of full likelihood coalescent modeling produced more robust results (Thawornwattana et al. 2021), but included only six out of 20 species in the SEC clade, making it impossible to infer the exact sources of admixture. The representation of intraspecific variation is also important: in our study we sample only the nonmimetic *H. hermathena hermathena* and thus cannot replicate the results of Massardo et al. (2020), who discovered introgression at *cortex* between *H. erato* and its mimic *H. hermathena vereatta*. Despite the difficulties in matching sufficient data with robust analytical tools, all approaches used in our and other studies point to *H. hecalesia* as a product of hybridization.

### Neither Concatenation nor Coalescent Trees Adequately Represent Species History

There has been a marked shift over recent years away from phylogenetic methods that involve concatenation of data, and toward approaches that involve coalescent modeling. Methods for inferring a species tree by modeling the incomplete sorting of loci represent an improvement on the assumption that there is a common evolutionary history across all genomic regions (Heled and Drummond 2010; Liu et al. 2010; Mirarab et al. 2014), although the variation in results demonstrates the need for better analytical tools, as well as more complete data. Across the tree of life, from birds (Reddy et al. 2017) and mammals (Chen et al. 2017) to land plants (Zhong et al. 2013) and fungi (Shen et al. 2016), treatment of individual gene trees under MSC methods has yielded substantially different results to simple concatenation. In contrast, our *Heliconius* trees are consistent with previous work (Beltran et al. 2007; Kozak et al. 2015; Zhang et al. 2016; Edelman et al. 2019), but clarify some uncertainties, including the placement of the *H. hecuba* and *H. egeria* groups, relationships in the *H. sapho* clade, and the position of *H. besckei*. Nonetheless, similar to other large phylogenetic studies (Brawand et al. 2014; Fontaine et al. 2015), none of the individual gene trees showed exactly the same topology as the autosomal MP-EST species tree, suggesting that the well-supported bifurcating trees do not fully represent the underlying signal in the genomes within this clade. Network modeling clearly demonstrates that introgression has been important throughout the evolution of the genus, and yet this process could easily be overlooked with many of the modern phylogenomic methods.

The comprehensive analysis of the large butterfly genus shows the important role of adaptive introgression at multiple loci in shaping radiations. The main appeal of studying adaptive radiations is their power for analyzing trait evolution in a comparative framework, and a growing number of studies are looking at several *Heliconius* characters through this lens (e.g., Briscoe et al. 2013; Sculfort et al. 2020). It is increasingly clear that many key adaptive traits are determined by introgressed sequences, and thus a comparative approach reliant on a single bifurcating species tree would give highly incomplete results (Hahn and Nakhleh 2016; Bastide et al. 2018). To expose the hidden uncertainties of phylogeny and capture the potential of adaptations to be shared between species, future work must utilize approaches that reflect the nonbifurcating reality of evolving genomes.

## Materials and Methods

### Sampling

*Heliconius* can be divided into two deep lineages (fig. 1 and supplementary file 1, Supplementary Material online). The first consists of *H. erato, H. sara, H. clysonymus* and relatives, and throughout the text we refer to this group as *SEC*. This lineage can be subdivided into three smaller clades of species closely related to *H. sara*, *H. erato*, and *H. clysonymus*. The clade of *H. clysonymus*, *H. hortense*, and *H. telesiphe* turned to be of special interest and we refer to it as *CHT*. The second major lineage within the genus are species related to *H. melpomene*, divided into five groups: the *H. melpomene/H. cydno* group; *H. numata* and relatives, often called “silvaniforms”; and the clades of *H. doris*, *H. wallacei*, and *H. aoede*. The first two clades are often grouped together and referred to as the *H. melpomene/cydno/silvaniform* clade (*MCS*).

We sampled 40 out of 47 *Heliconius*, as well six of the 12 species in the sister genus *Eueides*, and the monotypic *Dryadula* and *Agraulis* as outgroups (supplementary file 1, Supplementary Material online). Genomes of 11 species were re-sequenced for the first time: *Heliconius atthis*, *Heliconiusantiochus*, *Heliconiusegeria*, *Heliconiusleucadia*, *Heliconiusperuvianus, Eueides aliphera*, *Eueideslampeto*, *Eueideslineata*, *Eueidesisabella*, *Eueidesvibilia, Agraulis vanillae.* Material of sufficient quality could not be obtained for the remaining seven species of *Heliconius* and six of *Eueides*. Data for the other 37 species included in the study were published previously (Heliconius Genome Consortium 2012; Briscoe et al. 2013; Kronforst et al. 2013; Martin et al. 2013; Supple et al. 2013; Nadeau et al. 2016; Wallbank et al. 2016; Enciso-Romero et al. 2017; Jay et al. 2018). To enhance coalescent modeling by sampling genetic diversity (Edwards et al. 2016), we included individuals from distant populations and diverse wing pattern races when possible. Our full data set totaled 145 individuals and included multiple individuals of most species.

### DNA Sequencing

All sequencing data used in this study, novel and previously published, were generated with the Illumina technology with 100 bp paired-end reads, insert sizes of 250–500 bp and read coverage from 12× to 110×. In case of the new samples, DNA was extracted with the DNeasy Blood and Tissue kit (Qiagen) from 30 to 50 μg of thorax tissue homogenized in buffer ATL using the TissueLyser (Qiagen); purified by digesting with RnaseA (Qiagen); and quantified on a Qubit v.1 spectrophotometer (LifeTechnologies). Whole genome libraries with an average insert size of 500 bp were sequenced on a HiSeq 2500 to a mean coverage depth of 50.7× (range: 33.1–67.8×).

### Read Mapping and Genotyping

Raw reads were checked using FastQC v0.11 (Andrews 2014) and aligned to the *H. melpomene melpomene* reference v1 (Heliconius Genome Consortium 2012) with BWA v6 (Li and Durbin 2009). Initial BWA alignments were improved with Stampy v1.0.18 (Lunter and Goodson 2011). Aligner parameters were based on earlier empirical tests (Davey 2013; Nadeau et al. 2013) and the age of divergence from the reference (Kozak et al. 2015) (supplementary table 2, Supplementary Material online). Alignments were sorted with Samtools (Li et al. 2009), deduplicated with Picard v1.112 (Fennell 2010) and re-aligned in Genome Analysis Toolkit v3.1 (GATK) (McKenna et al. 2010; DePristo et al. 2011). SNPs were called separately across samples at sites with coverage >4× and quality >20 using the GATK Unified Genotyper (van der Auwera et al. 2013). Species genotypes were merged using Bcftools v1 (Li et al. 2009) and assessed with an in-house Python script (Martin et al. 2013) (evaluateVCF-03.py [Martin 2017]). We identified 126,865,683 individual SNPs (supplementary table 3, Supplementary Material online), including 5,483,419 in the exome. The autosomal matrix of exonic, biallelic, nonsingleton SNPs genotyped in all individuals contained 122,913 variants. Commands for genotyping and phylogenetic software are given in the Supplementary Methods.

### Exome Alignments and Gene Trees

Protein-coding genes can be effectively treated as discrete markers for multilocus phylogenetics (Edwards et al. 2016). Exonic markers were chosen over noncoding loci because 1) reads from distantly related species map better at the CDS; 2) orthologous sequences can be identified with greater confidence. We minimized paralogy by narrowing the gene set to 1:1:1 orthologs between *H. melpomene*, *Danaus plexippus*, and *Bombyx mori* identified by OrthoMCL (Li et al. 2003; Heliconius Genome Consortium 2012). Alignments of entire protein-coding, single-copy genes were extracted with an in-house script (gene_fasta_from_reseq.py [Martin 2017]). For this analysis, we ignored all genes within scaffolds linked to the color pattern loci (see table 2), as the exact boundaries of these loci have not been established for most species (Van Belleghem et al. 2017) and linkage to loci involved in adaptive color pattern differences might mislead phylogenetic inference.

We trimmed the alignments with TrimAl v1.2 (Capella-Gutiérrez et al. 2009), removing any sequences that contained >50% missing data (see the command line in Supplementary Methods). Furthermore, high entropy sections of each alignment were excluded by Block Mapping and Gathering with Entropy (BMGE) (Criscuolo and Gribaldo 2010) with a moderately relaxed PAM100 similarity matrix. Individual ML gene trees were estimated in FastTree v2.1 (Price et al. 2010) with parametric aLRT nodal support (Anisimova and Gascuel 2006). Species tree and network analyses listed below were conducted using rooted gene trees inferred from the 6,725 autosomal and 406 sex-linked CDS genes.

### Incongruence in the Data

To assess how much the topologies of gene trees differ from one another, we calculated the Robinson–Foulds distance (RF) (Robinson and Foulds 1981) for all pairs of trees, normalized by dividing the observed distance by the maximum possible RF between the two trees. This statistic was calculated using PAUP* v4 (Swofford 2002) across the entire data set of 145 samples and 6725 genes. As some of the differences are expected to arise from the lack of intraspecific resolution, the calculation was repeated on a thinned data set of 57 high coverage individuals representing all species, with additional individuals included in species with strong geographic structure (supplementary file 1, Supplementary Material online).

We identified highly incongruent nodes by computing 50% majority rule trees and using four information-theory measures (Salichos and Rokas 2013; Salichos et al. 2014) on the reduced data set of 57 samples at 6,725 genes. These analyzes were performed in RAxML v8 (Stamatakis 2014). The information-theory measures included the: 1) IC, which compares the frequency of a bipartition to the frequency of the most common alternative; 2) the IC All (ICA), which considers all alternatives with support ≥ 5%; 3) the corresponding tree certainty (TC), calculated and normalized over all bipartitions: and 4) the tree certainty All (TCA) (Salichos et al. 2014). All four measures are expressed on a scale from –1 (when the bipartition is not found in any of the gene trees) to 1 (when the bipartition is found in all). Because various patterns of missing data can impact the IC/TC values (Salichos et al. 2014), and many of our gene trees are incompletely resolved, we examined the impact of input data quality on these scores by repeating the procedure with the 1,000 most resolved phylogenies.

### Species Trees

Naïve “total evidence” phylogenies were estimated from concatenated exonic SNPs in RAxML v8 with 100 bootstrap replicates, GTR+Γ model and ascertainment bias correction (Stamatakis 2014). The history of the matriline was approximated from the whole-mitochondrial alignment with partitions determined by PartitionFinder v1.1 (Lanfear et al. 2012). In addition, we used two MSC approaches to estimate the species tree under the assumption of Incomplete Lineage Sorting. MP-EST v1.4 maximizes a pseudo-likelihood function over the distribution of taxon triples extracted from gene tree topologies, and provides a measure of incongruence based on the proportion of triples shared by the gene trees, similar to the RF distance (Liu et al. 2010). An MSC phylogeny was inferred in MP-EST from the 6,725 gene trees, evaluating support by re-estimating the tree 100 times with random samples of 500 gene trees. Since MP-EST may be misled by errors in gene tree reconstruction (Mirarab and Warnow 2015), we compared the results with ASTRAL-III, a fast quartet method that accounts for polytomies and low support values in the input (Zhang et al. 2017). ASTRAL quantifies discordance by computing how many of the gene trees contain the quartets making up the species tree (Sayyari and Mirarab 2016).

Recombination between exons scattered across a long genomic interval may lead to conflicting signals, which could be obscured by performing phylogenetic inference at the level of a complete coding gene, a practice criticized as “concatalescence” (Gatesy and Springer 2013). To account for this possibility, we repeated the ASTRAL-III species tree inference using individual autosomal exons longer than 500 base pairs. By restricting analysis to these 6,367 longer exons, we ensured sufficient information content, while eliminating the possibility that recombination between exons of one gene was responsible for conflicting signals.

### Admixture Networks

Following the identification of problematic nodes based on gene tree statistics, information criteria and conflicting nodes in species trees, we applied two distinct network approaches (Hahn and Nakhleh 2016). First, we determined the admixture graph (AG) in TreeMix v1.13 by identifying the pairs of taxa sharing more than the expected proportion of allelic variation (Pickrell and Pritchard 2012). Individuals were again assigned to taxa, distinguishing major clades within well-represented species as separate lineages. Relations between taxa were inferred from allele frequency data computed in PLINK v1.9 (Chang et al. 2015), based on the matrix of autosomal SNPs. As TreeMix assumes individual SNPs to be represented across samples and independent, the original matrix was filtered to remove sites with <95% complete data. We identified and pruned sites that could be linked within species, using the pairwise linkage disequilibrium estimator in PLINK v1.9 with default settings.

Second, we modeled both hybridization and incomplete lineage sorting under the MSC network framework implemented in PhyloNet v3.5. Networks were computed under the MPL criterion from the 6725 rooted autosomal phylogenies, considering only nodes with support >0.8 (*-b 0.8*) and starting with the MP-EST species tree. To search for ancient admixtures between deeper branches of the tree, we conducted an analysis with one species from each of the seven *Heliconius* clades (*H. melpomene*, *H. numata*, *H. doris*, *H. wallacei*, *H. erato*, *H. telesiphe*, *H. aoede*) and *Eueides*, as a full run with all the samples was not feasible. The optimal network was determined by calculating the Bayesian information criterion from the maximum likelihood and the number of lineages, admixture edges and gene trees in each model (supplementary file 3, Supplementary Material online) (Yu et al. 2014).

### Gene Flow in the H. erato Lineage

Admixture has been studied only in a few species of the *SEC* clade (Edelman et al. 2019; Massardo et al. 2020). Here, we focused on the events involving *H. hecalesia, H. clysonymus* and cognates. The extent of genome-wide similarity between species clusters was illustrated with PCAs of variation in the matrix of autosomal SNPs, calculated for the *SEC* clade in the R package *adegenet* (Jombart and Ahmed 2011). To test for gene flow we calculated the *D* statistic (Durand et al. 2011), derived from the allelic configurations of two taxa, P_1_ and P_2_, their relative P_3_ and an outgroup. Under the null hypothesis, no admixture occurred between P_3_ and either P_1_ or P_2_. Under the alternative, P_3_ exchanged alleles with either P_1_ or P_2_, resulting in the excess of the corresponding allelic configuration. Significance of the result can be assessed by block jackknifing (Martin et al. 2015). Specific tests were conducted for gene flow between *H. hecalesia* (P_2_) and *H. clysonymus, H. hortense, H. telesiphe* or species from the *H. sara* clade (P_3_). The sister species P_1_ was either the allopatric *H. erato* from French Guiana, or the parapatric *H. erato* from Amazonia, and the outgroup was always *H. melpomene*.

To address the hypothesis of hybrid origin of *H. hecalesia* or the *H. clysonymus/H. hortense* pair explicitly, we modeled various scenarios of divergence with gene flow under the maximum likelihood criterion as implemented in PHRAPL (Jackson et al. 2017). PHRAPL is a maximum likelihood method to assess complex scenarios including lineage coalescence, gene flow and population growth. PHRAPL uses simulations to compare all possible scenarios under a range of parameter values sampled from a predefined grid, using gene tree topology as the summary statistic (Jackson et al. 2017). We fitted models of speciation history to four distinct triplets of taxa: 1) *H. erato* (Western and Andean populations), *H. hecalesia* and *H. clysonymus* (including the sister species *H. hortense*); 2) *H. telesiphe*, *H. clysonymus* and *H. charithonia* (including the sister species *H. peruvianus*); 3) *H. erato*, *H. hecalesia* and *H. telesiphe*; 4) *H. telesiphe*, *H. charithonia* and *H. sara*. The four sets of taxa were chosen based on the earlier evidence for introgression from either MPL networks and TreeMix (1, 2) or the *D* statistics (1–4). To diminish the computational burden, we subsampled up to three tips per species from the 6,725 gene trees with 30 replicates per tree (Jackson et al. 2017). For each triplet, we evaluated a total of 48 models, including parameters for coalescence, symmetric migration, and change in population size between lineages, estimated at the default values for a PHRAPL grid search. The optimal model was selected by weighted Akaike’s information criterion (wAIC).

### Introgression at the Color Pattern Loci

The history of the loci associated with aposematic wing phenotypes is distinct due to strong selection (Möst et al. 2020), and thus the seven scaffolds in the *Hmel v1* assembly containing these loci (table 2) were treated separately. Each scaffold alignment was partitioned into windows of 20 kb, sliding by 10 kb, discarding windows with <1000 polymorphic sites [script sliPhy3.py (Martin 2017)]. The topology for every window was reconstructed and tested for significant differences from the MP-EST species tree using the SH test (Shimodaira and Hasegawa 1999) in RAxML. In order to understand precisely how the *Hmel v1* reference corresponds to the specific red control loci of *H. erato* (Supple et al. 2013), the *optix* (*B/D*) scaffold HE670865 was aligned against the *H. erato B/D* BACs (Papa et al. 2008) with mLAGAN (Brudno et al. 2003).

After finding discordant topologies at the *optix*, *cortex*, and *WntA* loci, we tested if the variation can be explained by incomplete lineage sorting alone, or whether gene flow in the *SEC* clade needs to be taken into account. We focused on specific intervals around the key genes, where departures from the species tree were found for *SEC*: HE670865:310,000:460,000, containing the *optix* protein CDS and several regulatory elements (Supple et al. 2013; Van Belleghem et al. 2017); HE667780:570000:750000, including *cortex* (Nadeau et al. 2016; Van Belleghem et al. 2017) and the novel inversion identified in some species of the *SEC* clade (Edelman et al. 2019); HE668478:450,000:500,000, containing *WntA* (table 2).

We analyzed all three genomic intervals separately under coalescent models in BEAST2. In each case, the interval was divided into windows of 10 kb treated as separate partitions, and the alignments were reduced to the relevant species: *H. hecalesia*; the *CHT* clade; *H. sara* and *H. charithonia*; *H. erato*, *H. himera* and *H. hermathena*. First, the coalescent tree model, where incongruence is purely due to incomplete lineage sorting, was fitted in StarBEAST2 (Ogilvie et al. 2017). Each partition was assigned the HKY+Γ substitution model with four rate categories, and a speciation-only tree model was selected for compatibility with the species network analysis. Based on the age of the *Eueides-Heliconius* split (Chazot et al. 2019), we used a strict molecular clock with an estimated exome-wide rate of 0.003 substitutions per site per MY. Two independent chains of 200 million cycles with 50% burnin were executed for each analysis; possible bias in priors was assessed by executing empty prior runs; and the convergence and effective sample sizes of the numeric parameters were visualized in Tracer (Rambaut et al. 2014). Second, the coalescent network was estimated in BEAST2 (Zhang et al. 2018): a model where incongruence can be a result of either ILS or admixture between species. Additional priors included: species net diversification rate (exponential with mean 1.0, corresponding to doubling every 1 My) and turnover rate (beta distribution: *α* = 3.0; *β* = 1.0, corresponding to low probability of admixture). The relative fit of the tree and network models to the same data was estimated by computing Bayes Factors from marginal likelihoods determined by Path Sampling with 20 steps and chains of 10 million cycles (Baele et al. 2013). Networks were visualized in IcyTree (Vaughan 2017).

## Supplementary Material

Supplementary data are available at *Genome Biology and Evolution* online.

## Supplementary Material

evab099_Supplementary_Data

## References

[evab099-B1] Andrews S. 2014. FastQC. http://www.bioinformatics.babraham.ac.uk/projects/fastqc/. [accessed 2014 July 1]

[evab099-B2] Anisimova M Gascuel O. 2006. Approximate likelihood-ratio test for branches: a fast, accurate, and powerful alternative. Syst Biol. 55(4):539–552. 16785212 10.1080/10635150600755453

[evab099-B3] Baele G Li WLS Drummond AJ Suchard MA Lemey P. 2013. Accurate model selection of relaxed molecular clocks in Bayesian phylogenetics. Mol Biol Evol. 30(2):239–243. 23090976 10.1093/molbev/mss243 PMC3548314

[evab099-B4] Bastide P Solís-Lemus C Kriebel R William Sparks K Ané C. 2018. Phylogenetic comparative methods on phylogenetic networks with reticulations. Syst Biol. 67(5):800–820. 29701821 10.1093/sysbio/syy033

[evab099-B5] Beltran M , et al 2007. Do pollen feeding, pupal-mating and larval gregariousness have a single origin in Heliconius butterflies? Inferences from multilocus DNA sequence data. Proc R Soc B. 92:221–239.

[evab099-B6] Brawand D , et al 2014. The genomic substrate for adaptive radiation in African cichlid fish. Nature 513(7518):375–381. 25186727 10.1038/nature13726 PMC4353498

[evab099-B7] Briscoe AD , et al 2013. Female behaviour drives expression and evolution of gustatory receptors in butterflies. PLoS Genet. 9(7):e1003620. 23950722 10.1371/journal.pgen.1003620 PMC3732137

[evab099-B8] Brower AVZ Garzón-Orduña IJ. 2018. Missing data, clade support and “reticulation”: the molecular systematics of Heliconius and related genera (Lepidoptera: Nymphalidae) re-examined. Cladistics 34(2):151–166. 10.1111/cla.12198 34645081

[evab099-B9] Brudno M , et al 2003. LAGAN and Multi-LAGAN: efficient tools for large-scale multiple alignment of genomic DNA. Genome Res. 13(4):721–731. 12654723 10.1101/gr.926603 PMC430158

[evab099-B10] Capella-Gutiérrez S Silla-Martínez JM Gabaldón T. 2009. trimAl: a tool for automated alignment trimming in large-scale phylogenetic analyses. Bioinformatics 25(15):1972–1973. 19505945 10.1093/bioinformatics/btp348 PMC2712344

[evab099-B11] Chamberlain NL Hill RI Baxter SW Jiggins CD Kronforst MR. 2011. Comparative population genetics of a mimicry locus among hybridizing *Heliconius* butterfly species. Heredity (Edinb). 107(3):200–204. 21304546 10.1038/hdy.2011.3 PMC3119732

[evab099-B12] Chang CC , et al 2015. Second-generation PLINK: rising to the challenge of larger and richer datasets. Gigascience 4:7. 25722852 10.1186/s13742-015-0047-8 PMC4342193

[evab099-B13] Chazot N , et al 2019. Priors and posteriors in Bayesian timing of divergence analyses: the age of butterflies revisited. Syst Biol. 68(5):797–813. 30690622 10.1093/sysbio/syz002 PMC6893297

[evab099-B14] Chen M-Y Liang D Zhang P. 2017. Phylogenomic resolution of the phylogeny of Laurasiatherian mammals: exploring phylogenetic signals within coding and noncoding sequences. Genome Biol Evol. 9(8):1998–2012. 28830116 10.1093/gbe/evx147 PMC5737624

[evab099-B15] Concha C , et al 2019. Interplay between developmental flexibility and determinism in the evolution of mimetic *Heliconius* wing patterns. Curr Biol. 29(23):3996–4009. 31735676 10.1016/j.cub.2019.10.010

[evab099-B16] Criscuolo A Gribaldo S. 2010. BMGE (Block Mapping and Gathering with Entropy): a new software for selection of phylogenetic informative regions from multiple sequence alignments. BMC Evol Biol. 10:210. 20626897 10.1186/1471-2148-10-210 PMC3017758

[evab099-B17] Cui R , et al 2013. Phylogenomics reveals extensive reticulate evolution in *Xiphophorus* fishes. Evolution 67(8):2166–2179. 23888843 10.1111/evo.12099

[evab099-B18] Dasmahapatra KK , et al 2007. Genetic analysis of a wild-caught hybrid between non-sister *Heliconius* butterfly species. Biol Lett. 3(6):660–663. 17804337 10.1098/rsbl.2007.0401 PMC2391228

[evab099-B19] Davey JW. 2013. heliconius.org: Aligning Heliconius short read sequences. [accessed 2013 May 1]. Available from: http://www.heliconius.org/2013/aligning-heliconius-short-read-sequences/.

[evab099-B20] Degnan JH. 2018. Modeling hybridization under the network multispecies coalescent. Syst Biol. 67(5):786–799. 29846734 10.1093/sysbio/syy040 PMC6101600

[evab099-B21] DePristo MA , et al 2011. A framework for variation discovery and genotyping using next-generation DNA sequencing data. Nat Genet. 43(5):491–498. 21478889 10.1038/ng.806 PMC3083463

[evab099-B22] Durand EY Patterson N Reich D Slatkin M. 2011. Testing for ancient admixture between closely related populations. Mol Biol Evol. 28(8):2239–2252. 21325092 10.1093/molbev/msr048 PMC3144383

[evab099-B23] Edelman NB , et al 2019. Genomic architecture and introgression shape a butterfly radiation. Science 366(6465):594–599. 31672890 10.1126/science.aaw2090 PMC7197882

[evab099-B24] Edwards SV Potter S Schmitt CJ Bragg JG Moritz C. 2016. Reticulation, divergence, and the phylogeography–phylogenetics continuum. Proc Natl Acad Sci USA. 113(29):8025–8032. 27432956 10.1073/pnas.1601066113 PMC4961137

[evab099-B25] Enciso-Romero J , et al 2017. Evolution of novel mimicry rings facilitated by adaptive introgression in tropical butterflies. Mol Ecol. 26(19):5160–5172. 28777894 10.1111/mec.14277

[evab099-B26] Feliner GN , et al 2017. Is homoploid hybrid speciation that rare? An empiricist’s view. Heredity 118:513–516. 28295029 10.1038/hdy.2017.7 PMC5436029

[evab099-B27] Fennell T. 2010. Picard Tools. broadinstitute.github.io/picard. [Accessed 2014 July 1]

[evab099-B28] Fontaine MC , et al 2015. Extensive introgression in a malaria vector species complex revealed by phylogenomics. Science 347(6217):1258524. 25431491 10.1126/science.1258524 PMC4380269

[evab099-B29] Gatesy J Springer MS. 2013. Concatenation versus coalescence versus ‘concatalescence’. Proc Natl Acad Sci USA. 110(13):E1179. 23460702 10.1073/pnas.1221121110 PMC3612616

[evab099-B18696471] Giska I , et al 2019. Introgression drives repeated evolution of winter coat color polymorphism in hares. Proc Natl Acad Sci U S A. 116(48):24150–24156. 31712446 10.1073/pnas.1910471116 PMC6883779

[evab099-B30] Gokcumen O. 2020. Archaic hominin introgression into modern human genomes. Yearbook Phys Anthropol. 171(S70):60–73. 10.1002/ajpa.23951 31702050

[evab099-B31] Hahn MW Nakhleh L. 2016. Irrational exuberance for resolved species trees. Evolution 70(1):7–17. 26639662 10.1111/evo.12832

[evab099-B32] Heled J Drummond AJ. 2010. Bayesian inference of species trees from multilocus data. Mol Biol Evol. 27(3):570–580. 19906793 10.1093/molbev/msp274 PMC2822290

[evab099-B33] Heliconius Genome Consortium. 2012. Islands of divergence underlie adaptive radiation in a butterfly genome. Nature 487:94–98.22722851

[evab099-B34] Hilario E Gogarten JP. 1993. Horizontal transfer of ATPase genes—the tree of life becomes a net of life. Biosystems 31(2–3):111–119. 8155843 10.1016/0303-2647(93)90038-e

[evab099-B35] Huerta-Sánchez E , et al 2014. Altitude adaptation in Tibetans caused by introgression of Denisovan-like DNA. Nature 512:194–197. 25043035 10.1038/nature13408 PMC4134395

[evab099-B36] Jackson ND Morales AE Carstens BC O’Meara BC. 2017. PHRAPL: phylogeographic inference using approximate likelihoods. Syst Biol. 66(6):1045–1053. 28204782 10.1093/sysbio/syx001

[evab099-B37] Jay P , et al 2018. Supergene evolution triggered by the introgression of a chromosomal inversion. Curr Biol. 28(11):1839–1845.e3. 29804810 10.1016/j.cub.2018.04.072

[evab099-B38] Jombart T Ahmed I. 2011. adegenet 1.3-1: new tools for the analysis of genome-wide SNP data. Bioinformatics 27(21):3070–3071. 21926124 10.1093/bioinformatics/btr521 PMC3198581

[evab099-B39] Joron M , et al 2006. A conserved supergene locus controls colour pattern diversity in *Heliconius* butterflies. PLoS Biol. 4(10):e303. 17002517 10.1371/journal.pbio.0040303 PMC1570757

[evab099-B40] Joron M , et al 2011. Chromosomal rearrangements maintain a polymorphic supergene controlling butterfly mimicry. Nature 477(7363):203–206. 21841803 10.1038/nature10341 PMC3717454

[evab099-B41] Kang JH Schartl M Walter RB Meyer A. 2013. Comprehensive phylogenetic analysis of all species of swordtails and platies (Pisces: genus *Xiphophorus*) uncovers a hybrid origin of a swordtail fish, *Xiphophorus monticolus*. BMC Evol Biol. 13:25. 23360326 10.1186/1471-2148-13-25 PMC3585855

[evab099-B42] Kozak KM , et al 2015. Multilocus species trees show the recent adaptive radiation of the mimetic heliconius butterflies. Syst Biol. 64(3):505–524. 25634098 10.1093/sysbio/syv007 PMC4395847

[evab099-B43] Kronforst MR , et al 2013. Hybridization reveals the evolving genomic architecture of speciation. Cell Rep. 5(3):666–677. 24183670 10.1016/j.celrep.2013.09.042 PMC4388300

[evab099-B44] Lamichhaney S , et al 2015. Evolution of Darwin’s finches and their beaks revealed by genome sequencing. Nature 518(7539):371–375. 25686609 10.1038/nature14181

[evab099-B45] Lanfear R Calcott B Ho SYW Guindon S. 2012. Partitionfinder: combined selection of partitioning schemes and substitution models for phylogenetic analyses. Mol Biol Evol. 29(6):1695–1701. 22319168 10.1093/molbev/mss020

[evab099-B46] Li H Durbin R. 2009. Fast and accurate short read alignment with Burrows–Wheeler transform. Bioinformatics 25(14):1754–1760. 19451168 10.1093/bioinformatics/btp324 PMC2705234

[evab099-B47] Li H , et al 2009. The Sequence Alignment/Map format and SAMtools. Bioinformatics 25(16):2078–2079. 19505943 10.1093/bioinformatics/btp352 PMC2723002

[evab099-B48] Li L Stoeckert CJ Roos DS. 2003. OrthoMCL: identification of ortholog groups for eukaryotic genomes. Genome Res. 13(9):2178–2189. 12952885 10.1101/gr.1224503 PMC403725

[evab099-B49] Liu L Yu L Edwards SV. 2010. A maximum pseudo-likelihood approach for estimating species trees under the coalescent model. BMC Evol Biol. 10:302. 20937096 10.1186/1471-2148-10-302 PMC2976751

[evab099-B50] Lunter G Goodson M. 2011. Stampy: a statistical algorithm for sensitive and fast mapping of Illumina sequence reads. Genome Res. 21(6):936–939. 20980556 10.1101/gr.111120.110 PMC3106326

[evab099-B51] Mallet J Beltran M Neukirchen W Linares M. 2007. Natural hybridization in heliconiine butterflies: the species boundary as a continuum. BMC Evol Biol. 7:28. 17319954 10.1186/1471-2148-7-28 PMC1821009

[evab099-B52] Martin A , et al 2012. Diversification of complex butterfly wing patterns by repeated regulatory evolution of a *Wnt* ligand. Proc Natl Acad Sci USA. 109(31):12632–12637. 22802635 10.1073/pnas.1204800109 PMC3411988

[evab099-B53] Martin SH. 2017. Genomics general scripts. https://github.com/simonhmartin. [Accessed 2019 March 1]

[evab099-B54] Martin SH Davey JW Jiggins CD. 2015. Evaluating the use of ABBA-BABA statistics to locate introgressed loci. Mol Biol Evol. 32(1):244–257. 25246699 10.1093/molbev/msu269 PMC4271521

[evab099-B55] Martin SH , et al 2013. Genome-wide evidence for speciation with gene flow in *Heliconius* butterflies. Genome Res. 23(11):1817–1828. 24045163 10.1101/gr.159426.113 PMC3814882

[evab099-B56] Massardo D , et al 2020. The roles of hybridization and habitat fragmentation in the evolution of Brazil’s enigmatic longwing butterflies, *Heliconius nattereri* and *H. hermathena*. BMC Biol. 18(1):84. 32620168 10.1186/s12915-020-00797-1 PMC7334841

[evab099-B57] Mazo-Vargas A , et al 2017. Macroevolutionary shifts of WntA function potentiate butterfly wing-pattern diversity. Proc Natl Acad Sci USA. 114(40):10701–10706. 28923954 10.1073/pnas.1708149114 PMC5635894

[evab099-B58] McKenna A , et al 2010. The Genome Analysis Toolkit: a MapReduce framework for analyzing next-generation DNA sequencing data. Genome Res. 20(9):1297–1303. 20644199 10.1101/gr.107524.110 PMC2928508

[evab099-B59] Mirarab S , et al 2014. ASTRAL: genome-scale coalescent-based species tree estimation. Bioinformatics 30(17):i541–8. 25161245 10.1093/bioinformatics/btu462 PMC4147915

[evab099-B60] Mirarab S Warnow T. 2015. ASTRAL-II: coalescent-based species tree estimation with many hundreds of taxa and thousands of genes. Bioinformatics 31(12):i44–i52. 26072508 10.1093/bioinformatics/btv234 PMC4765870

[evab099-B61] Moest M , et al. 2020. Selective sweeps on novel and introgressed variation shape mimicry loci in a butterfly adaptive radiation. PLoS Biol. 18(2):e3000597. 32027643 10.1371/journal.pbio.3000597 PMC7029882

[evab099-B62] Morris J , et al 2019. The genetic architecture of adaptation: convergence and pleiotropy in Heliconius wing pattern evolution. Heredity (Edinb). 123(2):138–152. 30670842 10.1038/s41437-018-0180-0 PMC6781118

[evab099-B63] Nadeau NJ , et al 2013. Genome-wide patterns of divergence and gene flow across a butterfly radiation. Mol Ecol. 22(3):814–826. 22924870 10.1111/j.1365-294X.2012.05730.x

[evab099-B64] Nadeau NJ , et al 2016. The gene cortex controls mimicry and crypsis in butterflies and moths. Nature 534(7605):106–110. 27251285 10.1038/nature17961 PMC5094491

[evab099-B65] Ogilvie HA Bouckaert RR Drummond AJ. 2017. StarBEAST2 brings faster species tree inference and accurate estimates of substitution rates. Mol Biol Evol. 34(8):2101–2114. 28431121 10.1093/molbev/msx126 PMC5850801

[evab099-B66] Papa R , et al 2008. Highly conserved gene order and numerous novel repetitive elements in genomic regions linked to wing pattern variation in *Heliconius* butterflies. BMC Genomics 9:345. 18647405 10.1186/1471-2164-9-345 PMC2515155

[evab099-B67] Pardo-Diaz C , et al 2012. Adaptive introgression across species boundaries in *Heliconius* butterflies. PLoS Genet. 8(6):e1002752. 22737081 10.1371/journal.pgen.1002752 PMC3380824

[evab099-B68] Pickrell JK Pritchard JK. 2012. Inference of population splits and mixtures from genome-wide allele frequency data. PLoS Genet. 8(11):e1002967. 23166502 10.1371/journal.pgen.1002967 PMC3499260

[evab099-B69] Price MN Dehal PS Arkin AP. 2010. FastTree 2–approximately maximum-likelihood trees for large alignments. PLoS One 5(3):e9490. 20224823 10.1371/journal.pone.0009490 PMC2835736

[evab099-B70] Rambaut A Suchard M Xie W Drummond A. 2014. Tracer v. 1.6. http://beast.bio.ed.ac.uk/.

[evab099-B71] Reddy S , et al 2017. Why do phylogenomic data sets yield conflicting trees? Data type influences the avian tree of life more than taxon sampling. Syst Biol. 66(5):857–879. 28369655 10.1093/sysbio/syx041

[evab099-B72] Reed RD , et al 2011. *Optix* drives the repeated convergent evolution of butterfly wing pattern mimicry. Science 333(6046):1137–1141. 21778360 10.1126/science.1208227

[evab099-B73] Robinson DF Foulds LR. 1981. Comparison of phylogenetic trees. Math Biosci. 53(1–2):131–147.

[evab099-B74] Rosser N Phillimore AB Huertas B Willmott KR Mallet J. 2012. Testing historical explanations for gradients in species richness in heliconiine butterflies of tropical America. Biol J Linn Soc. 105(3):479–497.

[evab099-B75] Roure B Baurain D Philippe H. 2013. Impact of missing data on phylogenies inferred from empirical phylogenomic data sets. Mol Biol Evol. 30(1):197–214. 22930702 10.1093/molbev/mss208

[evab099-B76] Salazar C , et al 2010. Genetic evidence for hybrid trait speciation in heliconius butterflies. PLoS Genet. 6(4):e1000930. 20442862 10.1371/journal.pgen.1000930 PMC2861694

[evab099-B77] Salazar C Jiggins CD Taylor JE Kronforst MR Linares M. 2008. Gene flow and the genealogical history of *Heliconius heurippa*. BMC Evol Biol. 8:132. 18454858 10.1186/1471-2148-8-132 PMC2391162

[evab099-B78] Salichos L Rokas A. 2013. Inferring ancient divergences requires genes with strong phylogenetic signals. Nature 497(7449):327–331. 23657258 10.1038/nature12130

[evab099-B79] Salichos L Stamatakis A Rokas A. 2014. Novel information theory-based measures for quantifying incongruence among phylogenetic trees. Mol Biol Evol. 31(5):1261–1271. 24509691 10.1093/molbev/msu061

[evab099-B80] Sayyari E Mirarab S. 2016. Fast coalescent-based computation of local branch support from quartet frequencies. Mol Biol Evol. 33(7):1654–1668. 27189547 10.1093/molbev/msw079 PMC4915361

[evab099-B81] Schumer M Rosenthal GG Andolfatto P. 2014. How common is homoploid hybrid speciation? Evolution 68(6):1553–1560. 24620775 10.1111/evo.12399

[evab099-B82] Sculfort O , et al 2020. Variation of chemical compounds in wild Heliconiini reveals ecological and historical contributions to the evolution of chemical defences in mimetic butterflies. Ecol Evol. doi:10.1002/ece3.6044. 10.1002/ece3.6044 PMC7069300 32185010

[evab099-B8446206] Seixas FA, Edelman NB, Mallet J. 2021. Synteny-based genome assembly for 16 species of Heliconius butterflies, and an assessment of structural variation across the genus. Genome Biology and Evolution. Accepted:10.1093/gbe/evab069 PMC8290116 33792688

[evab099-B83] Shen X-X , et al 2016. Reconstructing the backbone of the Saccharomycotina yeast phylogeny using genome-scale data. G3 Genes, Genomes, Genet. 6:3927–3939. 10.1534/g3.116.034744 PMC5144963 27672114

[evab099-B8527807] Sheppard PM, Turner JRG, Brown KS, Benson WW, Singer MC. 1985. Genetics and the evolution of muellerian mimicry in Heliconius butterflies. Philosophical Transactions of the Royal Society B. 308(1137):433–610.

[evab099-B84] Shimodaira H Hasegawa M. 1999. Multiple comparisons of log-likelihoods with applications to phylogenetic inference. DNA Seq. 16(8):1114–1116.

[evab099-B85] Stamatakis A. 2014. RAxML version 8: a tool for phylogenetic analysis and post-analysis of large phylogenies. Bioinformatics 30(9):1312–1313. 24451623 10.1093/bioinformatics/btu033 PMC3998144

[evab099-B86] Stryjewski KF Sorenson MD. 2017. Mosaic genome evolution in a recent and rapid avian radiation. Nat Ecol Evol. 1(12):1912–1922. 29085063 10.1038/s41559-017-0364-7

[evab099-B87] Supple MA , et al 2013. Genomic architecture of adaptive color pattern divergence and convergence in *Heliconius* butterflies. Genome Res. 23(8):1248–1257. 23674305 10.1101/gr.150615.112 PMC3730099

[evab099-B88] Swofford R. 2002. PAUP: Phylogenetic Analysis Using Parsimony (*and other methods). Available from: http://phylosolutions.com/paup-test. [Accessed 2016 June 1]

[evab099-B89] Thawornwattana Y , et al 2021. ‘Complex introgression history of the erato *–* sara clade of Heliconius butterflies’. bioRxiv 10.1101/2021.02.10.430600.

[evab099-B90] Van Belleghem SM , et al 2017. Complex modular architecture around a simple toolkit of wing pattern genes. Nat Evol Ecol. 1:1–32. 10.1038/s41559-016-0052 PMC5432014 28523290

[evab099-B91] van der Auwera GA , et al 2013. From fastq data to high-confidence variant calls: the Genome Analysis Toolkit best practices pipeline. Curr Protoc Bioinform. 43:1–33. 10.1002/0471250953.bi1110s43 PMC4243306 25431634

[evab099-B92] Vaughan TG. 2017. IcyTree: rapid browser-based visualization for phylogenetic trees and networks. Bioinformatics 33(15):2392–2394. 28407035 10.1093/bioinformatics/btx155 PMC5860111

[evab099-B93] Wallbank RWR , et al 2016. Evolutionary novelty in a butterfly wing pattern through enhancer shuffling. PLoS Biol. 14(1):e1002353. 26771987 10.1371/journal.pbio.1002353 PMC4714872

[evab099-B94] Wen D Yu Y Zhu J Nakhleh L. 2018. Inferring phylogenetic networks using PhyloNet. Syst Biol. 67(4):735–740. 29514307 10.1093/sysbio/syy015 PMC6005058

[evab099-B95] Westerman EL , et al 2018. Aristaless controls butterfly wing color variation used in mimicry and mate choice. Curr Biol. 28(21):3469–3474.e4. 30415702 10.1016/j.cub.2018.08.051 PMC6234856

[evab099-B96] Whitney KD Randell RA Rieseberg LH. 2010. Adaptive introgression of abiotic tolerance traits in the sunflower *Helianthus annuus*. New Phytol. 187(1):230–239. 20345635 10.1111/j.1469-8137.2010.03234.x

[evab099-B97] Wiens JJ Morrill MC. 2011. Missing data in phylogenetic analysis: reconciling results from simulations and empirical data. Syst Biol. 60(5):719–731. 21447483 10.1093/sysbio/syr025

[evab099-B98] Yu Y Dong J Liu KJ Nakhleh L. 2014. Maximum likelihood inference of reticulate evolutionary histories. Proc Natl Acad Sci USA. 111(46):11648–11653. 10.1073/pnas.1407950111 PMC4246314 25368173

[evab099-B99] Zhang C Ogilvie HA Drummond AJ Stadler T. 2018. Bayesian inference of species networks from multilocus sequence data. Mol Biol Evol. 35(2):504–517. 29220490 10.1093/molbev/msx307 PMC5850812

[evab099-B100] Zhang C Sayyari E Mirarab S. 2017. ASTRAL-III: increased scalability and impacts of contracting low support branches. In: RECOMB-CG 2017. Cham (Switzerland): Springer. p. 53–75.

[evab099-B101] Zhang W Dasmahapatra Kanchon K Mallet J Moreira GRP Kronforst MR. 2016. Genome-wide introgression among distantly related *Heliconius* butterfly species. Genome Biol. 17:25. 26921238 10.1186/s13059-016-0889-0 PMC4769579

[evab099-B102] Zhang W , et al 2019. Comparative transcriptomics provides insights into reticulate and adaptive evolution of a butterfly radiation. Genome Biol Evol. 11(10):2963–2975. 31518398 10.1093/gbe/evz202 PMC6821300

[evab099-B103] Zheng Y Janke A. 2018. Gene flow analysis method, the D-statistic, is robust in a wide parameter space. BMC Bioinformatics 19(1):10. 29310567 10.1186/s12859-017-2002-4 PMC5759368

[evab099-B104] Zhong B Liu L Yan Z Penny D. 2013. Origin of land plants using the multispecies coalescent model. Trends Plant Sci. 18(9):492–495. 23707196 10.1016/j.tplants.2013.04.009

