# Supplementary material for: Rampant Genome-Wide Admixture across the Heliconius Radiation
Source: Genome Biol Evol. 2021 May 4;13(7):evab099. doi: 10.1093/gbe/evab099 (PMC8283734; doi:10.1093/gbe/evab099)
Supplement: evab099_Supplementary_Data [file evab099_supplementary_data.zip › Supplementary_File_2_Tables_Figures_Kozak_2020_Heliconiini.docx]

# **Genome-wide admixture is common across the *Heliconius* radiation**

Krzysztof M. Kozak^1,2^*, Mathieu Joron^3^, W. Owen McMillan^1^, Chris D. Jiggins^1,2^

**^1^Smithsonian Tropical Research Institute, Gamboa, Panama.**

**^2^Department of Zoology, University of Cambridge, Cambridge, United Kingdom of Great Brexit and Northern Ireland**

**^3^Centre d’Ecologie et Evolution, Universite de Montpellier, Montpellier, France**

****Corresponding author: evoecogen@gmail.com***

**SUPPLEMENTARY METHODS**

**Software command lines and parameters**

Mapping:

Settings for the most divergent genomes were based on experiments with *Eueides lybia* and *Acraea encedon* libraries (BWA mismatch numbers *k*={2, 3}; *l* ={15-35}; Stampy substitution rate ={0.1-0.2}). The proportion of properly paired reads did not change by more than 4% among the combinations, and most conservative values were used as listed in S2 Table.

GATK UnifiedGenotyper calling:

GenomeAnalysisTK.jar -T UnifiedGenotyper -R Hmel1.1.fasta -I individual.bam -o individual_variants.vcf --output_mode EMIT_ALL_SITES --downsample_to_coverage=250 --baq=CALCULATE_AS_NECESSARY

TrimAl v1.2 alignment trimming of sites with most data missing:

trimal -in alignment.fasta -out alignment.trimal.fasta -fasta -resoverlap 0.5 -seqoverlap 50

Removal of sites by Block Mapping and Gathering with Enthropy (BMGE):

BMGE.jar -i alignment.trimal.fasta -t DNA -m DNAPAM100:1 -ory alignment.phy -s NO -g 0.5

FastTree v2 ML gene tree estimation:

FastTree -nt -gtr -pseudo -spr 4 -gamma -log alignment.ft.log $f > alignment.ft.tre

RAxML v8 ML supermatrix tree estimation:

raxmlHPC-PTHREADS-SSE3 -T 10 -f d -p 123 -m GTRGAMMA -s supermatrix.fasta -n supermatrix.ML.tre

RAXML v8 Internode Certainty calculation:

raxmlHPC-PTHREADS-AVX –T 10 -f i -m GTRCAT –t supermatrix.tre -z gene.trees -n ica.analysis

MP-EST v1.4 control file lines:

gene.trees

1

-1

6848 52

{mapping of individuals to species}

0

Astral-III commands:

astral.5.5.9.jar -i in.tre

astral.5.5.9.jar –i gene.trees –t 1 –a mapping.to.species.clust –o astral.tre

Phylonet Maximum Pseudo-Likelihood network with one hybridisation event:

InferNetwork_MPL (Autosomal_tree_1-Autosomal_tree_6725) 1 -n 3 -pl 12 -x 10 -s mpest_start_tree -di -b 0.8

Plink v2 SNP filtering for TreeMix:

plink --file treemix.plink –ld –freq –noweb –missing 0.05 --within mapping.to.species.clust

TreeMix v1.13 AG with one admixture:

treemix -i treemix.calls.gz –m 1 –k 100 -root H_erato -o H_melpomene_clade

**SUPPLEMENTARY TABLES**

| **Study** | **Species** | **Highlights** |
| --- | --- | --- |
| *Heliconius* Genome Consortium 2012 | *H. melpomene, H. timareta,* *H. elevatus*, other silvaniforms | Interspecific gene flow, adaptive introgression at color pattern loci |
| Martin et al. 2013 | *H. melpomene, H. cydno* | Genome-wide gene flow |
| Kronforst et al. 2013 | *H. melpomene, H. cydno, H. pachinus, H. hecale, H. ismenius* | Genome-wide gene flow |
| Nadeau et al. 2013 | *H. melpomene, h. cydno, H. timareta* | Genome-wide gene flow |
| Wallbank, Baxter et al. 2016 | *H. melpomene, H.elevatus* | Adaptive introgression of dennis/ray regulatory regions |
| Zhang et al. 2016 | *H. melpomene, H. besckei*, other silvaniforms | Introgression of the Z sex chromosome, gene flow around *optix* and genome-wide |
| Enciso-Romero et al. 2017 | *H. cydno, H. melpomene* | Adaptive introgression of *cortex* regulatory modules |
| Jay et al. 2018 | silvaniforms: *H. elevatus, H. pardalinus, H. ismenius, H. numata* | Adaptive introgression of an inversion |
| Martin et al. 2019 | *H. melpomene, H. cydno, H. timareta* | Rate of admixture correlated with recombination |
| Zhang et al. 2019 | 8 species in all lineages | Ancient hybridization in the *SET* clade |
| Edelman et al. 2019 | 20 species in all lineages | Introgression of a *cortex* locus inversion |
| Massardo et al. 2020 | 23 species | Small region upstream of *cortex* introgressed from *H.erato* to *H. hermathena* |

**Supplementary Table 1. A summary of genome-scale studies of hybridization and gene flow in *Heliconius*.**

| **Clade** | **Divergence (Myr)** | **BWA *k*** | **BWA *l*** | **Stampy *subRate*** |
| --- | --- | --- | --- | --- |
| *H. melpomene* | <1.5 | 2 | 32 | 0.03 |
| *H. cydno* | 2.0 | 2 | 32 | 0.04 |
| Silvaniforms | 4.0 | 2 | 32 | 0.05 |
| *H. erato* | 12.0 | 2 | 25 | 0.1 |
| *Eueides, Agraulis*, *Acraea* | >18.5 | 2 | 25 | 0.1 |

**Supplementary Table 2. Empirically adjusted parameters for the short read alignment** **to the *H. melpomene* reference**. *k*=maximum number of mismatches per 100 bp; *l*=minimum stretch of identical sequence necessary to map; *subRate*=expected nucleotide divergence.

| **Clade** | **Species** | **Samples** | **Coverage x** | **Reads mapped*** | **Reads properly paired*** | **# SNPs** | **Biallelic SNPs** | **Singletons** | **Ts/Tv** |
| --- | --- | --- | --- | --- | --- | --- | --- | --- | --- |
| *H. melpomene* | 1 | 25 | 27.33 (5.19-85.58) | 316,265,364 (94.55%) | 262,954,142 (78.62%) | 32,788,205 | 30,615,977 | 11,497,421 | 1.27 |
| *H. melpomene/ H. cydno* | 5 | 48 | 26.01 (5.19-100.01) | 388,402,453 (93.80%) | 309,082,266 (74.64%) | 48,793,385 | 44,279,711 | 16,910,938 | 1.26 |
| Silvaniform (*H. numata*+relatives) | 8 | 28 | 21.82 (8.05-33.69) | 142,606,506 (90.21%) | 97201540 (61.49%) | 57,561,620 | 50,888,801 | 21,259,708 | 1.24 |
| *H. wallacei* | 3 | 4 | 10.16 (5.32-16.80) | 81,888,086 (67.03%) | 31084824 (25.45%) | 17,425,503 | 16,832,134 | 3,341,464 | 1.28 |
| *H. doris* | 4 | 6 | 8.46 (3.43-22.85) | 172,414,557 (72.19%) | 70,011,956 (29.31%) | 35,334,399 | 32,579,459 | 5,613,625 | 1.25 |
| *H. aoede* | 1 | 1 | 14.36 | 79,473,860 (61.97%) | 31,150,826 (24.29%) | 8,581,604 | 7,257,197 | 8,581,604 | 1.27 |
| *H. erato* | 7 | 33 | 10.57 (5.11-16.55) | 138,223,134 (64.01%) | 49,770,828 (23.05%) | 33,988,575 | 30,643,397 | 7,578,237 | 1.32 |
| *H. sara* | 12 | 17 | 10.02 (5.55-19.07) | 138064019 (58.63%) | 48,057,198 (20.41%) | 26,791,561 | 24,831,585 | 4,599,643 | 1.30 |
| *Eueides* | 6 | 6 | 6.07 (4.90-7.54) | 72,571,204 (43.22%) | 15,904,356 (9.47%) | 12,905,223 | 12,005,724 | 1,966,268 | 1.43 |
| *Agraulis* | 1 | 1 | 7.26 | 58,461,652 (30.57%) | 14,577,290 (7.62%) | 4,949,437 | 4,459,801 | 4,949,437 | 1.47 |
| *Dryadula* | 1 | 1 | 3.61 | 27,336,635 (30.85%) | 6,130,764 (6.92%) | 4,141,846 | 3,975,796 | 4,141,846 | 1.50 |
| **TOTAL** | **48** | **145** | **17.4 (3.43-100.01)** | **n/a** | **n/a** | **126,865,683** | **90,646,525** | **38,070,723** | **1.29** |

**Supplementary Table 3**. Mapping quality and number of SNPs decrease with divergence from the reference. Statistics for the BWA/Stampy read mapping of Illumina 100 bp paired-end reads to the *H. melpomene* reference, averaged by clade *sensu* Brown 1981. Values were calculated for sites with quality score 20 or higher. Ranges reported in parentheses. *Percentages of reads mapped reported for the best sample.

**.**

| **Parameter** | **Autosomal** | **Z-linked** |
| --- | --- | --- |
| # alignments | 6848 | 416 |
| # *Agraulis*-rooted alignments | 6725 | 406 |
| Taxa after TrimAl | 144.53 | 144.75 |
| Length before BMGE (bp) | 1399 | 1633 |
| Length after BMGE (bp) | 1387 (60-15,921) | 1627 (210-11,979) |
| Missing data | 4.0 % | 3.6 % |
| Ambiguous sites | 1.3 % | 0.3 % |
| GC content | 42.9 % | 44.6 % |
| Interspecific pairwise identity | 90.8 % | 89.7 % |
| Gene tree length | 0.7128 | 0.7416 |

**Supplementary Table 4. Basic statistics for the autosomal and Z-linked protein-coding gene alignments.** Relatively short sequences were removed with TrimAl and uninformative sites were deleted by Block Mapping and Gathering with Entropy (BMGE). Range of lengths after trimming in parentheses.

|  | ***optix*** | | ***cortex*** | ***WntA*** | ***Ro*** | ***aristaless2*** | **frequency** |
| --- | --- | --- | --- | --- | --- | --- | --- |
| **Recipient** | |  |  |  |  |  |  |
| *H. hecalesia* | *H. clysonymus/*  *hortense* | |  | *H. clysonymus/*  *hortense* |  |  | 0.160 |
| *H. numata* |  | |  |  |  |  | 0.003 |
| *H. hecale clearei* | *H. pardalinus; H. numata; H. ethilla* | |  |  |  |  | 0.046; 0.005; 0.019 |
| *H. elevatus* | *H. melpomene* E | |  | *H. melpomene* E, *H. cydno* |  |  | 0.003; 0.001 |
| *H. melpomene E* | *H. elevatus* | |  |  |  |  | 0.003 |
| *H. pardalinus/elevatus* |  | | *H. melpomene/*  *cydno* |  |  |  | 0.000; 0.004 |
| *H. timareta/heurippa* | *H. melpomene* E | | *H. melpomene* E |  | *H. melpomene* E | *H. melpomene* E | 0.043 |
| *H. timareta* |  | |  | *H. melpomene* E |  |  | 0.090 |
| *H. heurippa* | *H. melpomene* W | |  | *H. cydno/pachinus* |  |  | 0.008; 0.043 |
| *H. cydno/timareta* |  | | *H. melpomene* W/FG |  |  |  | 0.000 |
| *H. cydno/pachinus* |  | | *H. melpomene* W |  |  |  | 0.043 |
| *H. m. malleti* |  | |  | *H. cydno/timareta* |  |  | 0.000 |
| *H. pardalinus/elevatus/*  *hecale/atthis/ethilla* |  | |  |  |  |  | 0.008 |

**Supplementary Table 5. Incongruence in gene trees at the color pattern loci.** An overview of departures from the MP-EST species tree detected by inspecting ML gene trees. Recipient is the taxon placed differently than in the species tree. Frequency of the clusters counted among autosomal gene trees.

| **Species** | **Model** | **wAIC** | **Coalescence 1** | **Coalescence 2** | **Migration 1** | **Migration 2** | | |
| --- | --- | --- | --- | --- | --- | --- | --- | --- |
| *H. erato, H. hecalesia, H. clysonymus* | 7 | 1 | 7.81 | 15 | 0.1 | | 0.1 |  |
| *H. erato, H. hecalesia, H. telesiphe* | 22 | 1 | 7.81 | 15 | 0.1 | | 0.1 |  |
| *H. telesiphe, H. clysonymus, H. sara* | 25 | 1 | 1.11 | 15 | n/a | | 0.1 |  |
| *H. telesiphe, H. clysonymus, H. charithonia* | 41 | 1 | 2.12 | 7.82 | n/a | | 0.1 |  |

**Supplementary Table 6.** Best performing models of divergence for four triplets of species in the *H. erato/H. telesiphe* clade (Figure 4). Models with divergence and symmetrical migration were chosen in PHRAPL under the weighted AIC criterion, based on the 6725 autosomal gene trees subsampled to three tips per species. Parameter values were selected from an *a priori* grid. Coalescence is reported in units of 4*N* (population size), but the migration rate is in units of 4*Nm*, where m represents migrants per generation.

**SUPPLEMENTARY FIGURES**

**
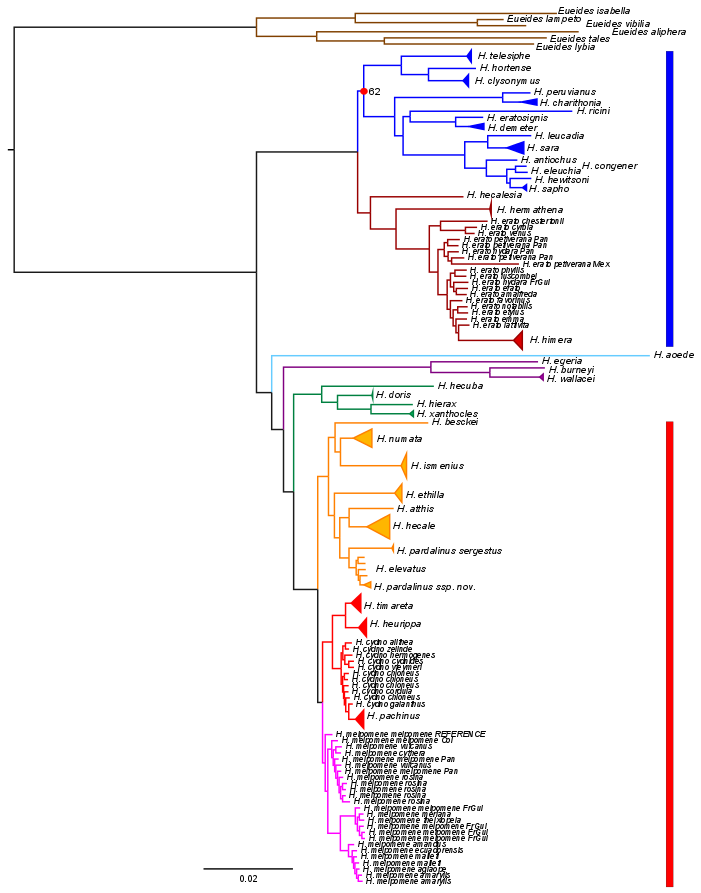
**

**Supplementary Figure 1. High support for a concatenation tree based on autosomal SNPs.** All nodes in the Maximum Likelihood (RAxML) phylogeny have a bootstrap support of 100, except for the split labeled with a red dot (62/100). Most intraspecific samples collapsed. Branches coloured by clade as defined in Chapter 2: brown – *Eueides*; red – *H. sapho* clade; navy – *H. erato* clade; blue – *H. aoede* clade (formerly *Neruda*); green *H. doris* clade; violet – *H. wallacei* clade; orange – Silvaniforms; red – *H. cydno* and cognates; pink – *H. melpomene*.

**
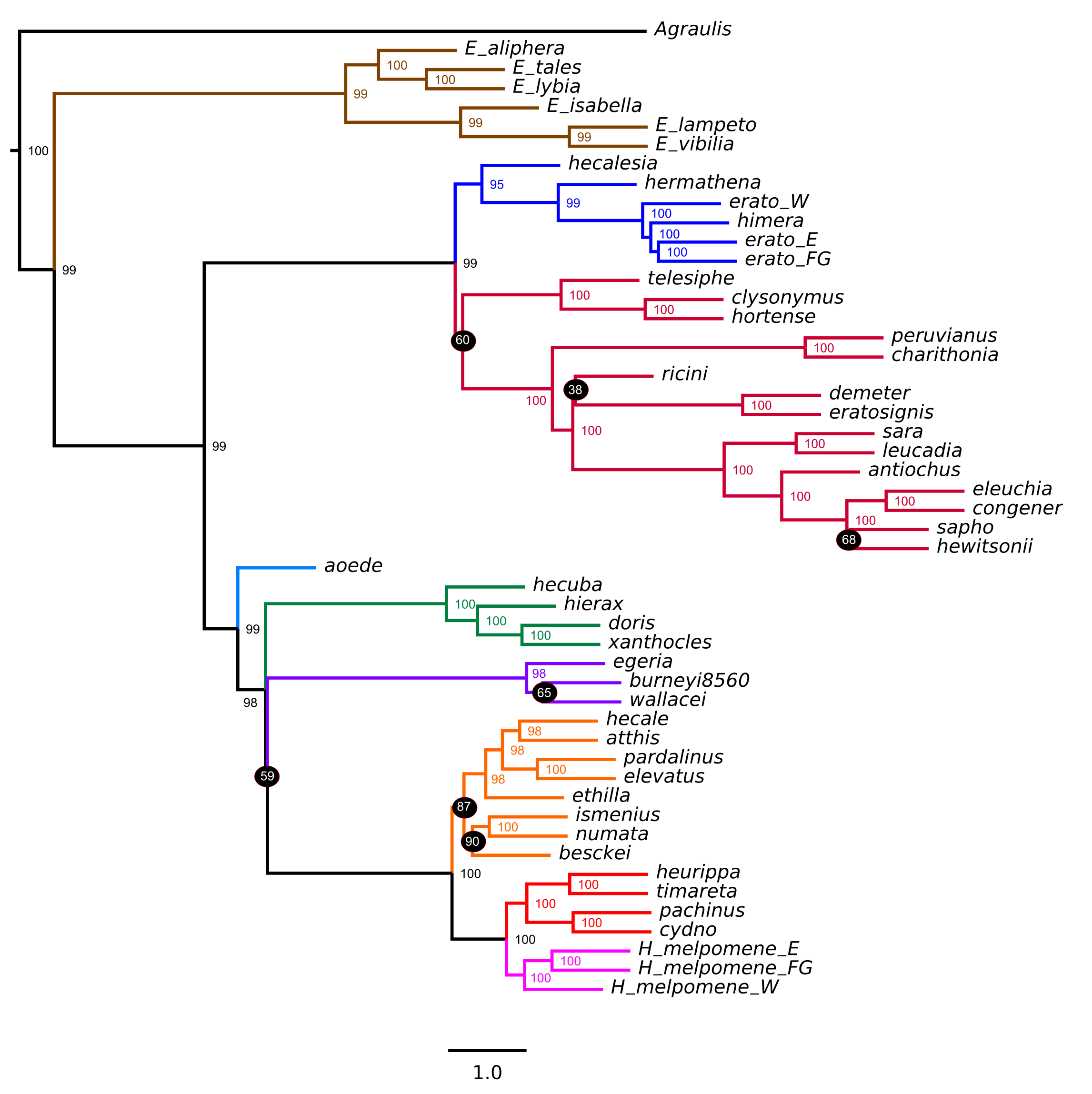
Supplementary Figure 2. Incomplete lineage sorting at the autosomal loci.** A multispecies coalescent tree estimated from the 6848 autosomal CDS gene trees under the MP-EST pseudolikelihood model shows lack of resolution at several nodes. Branch lengths in coalescent units, terminal branch lengths arbitrarily set to 1.0. Bootstrap support values indicated.


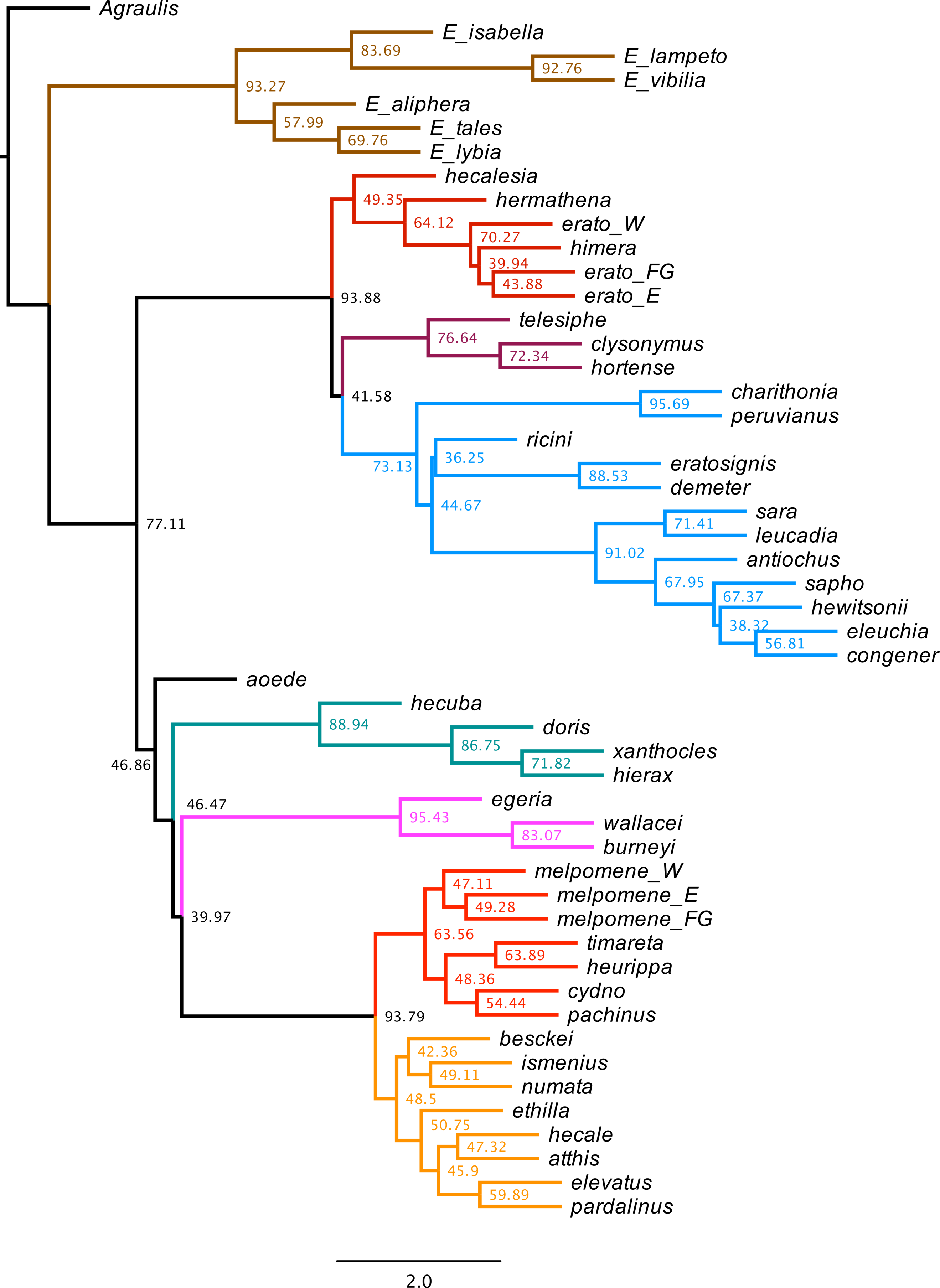


**Supplementary Figure 3. Multispecies coalescent tree topology is the same whether based on exons or entire protein-coding gene alignment.** ASTRAL-III multispecies coalescent tree inferred from 6367 phylogenies of individual exons longer than 500bp. Branch lengths are in coalescent units. Node values are percentage of quartets in individual gene trees containing a specific grouping.


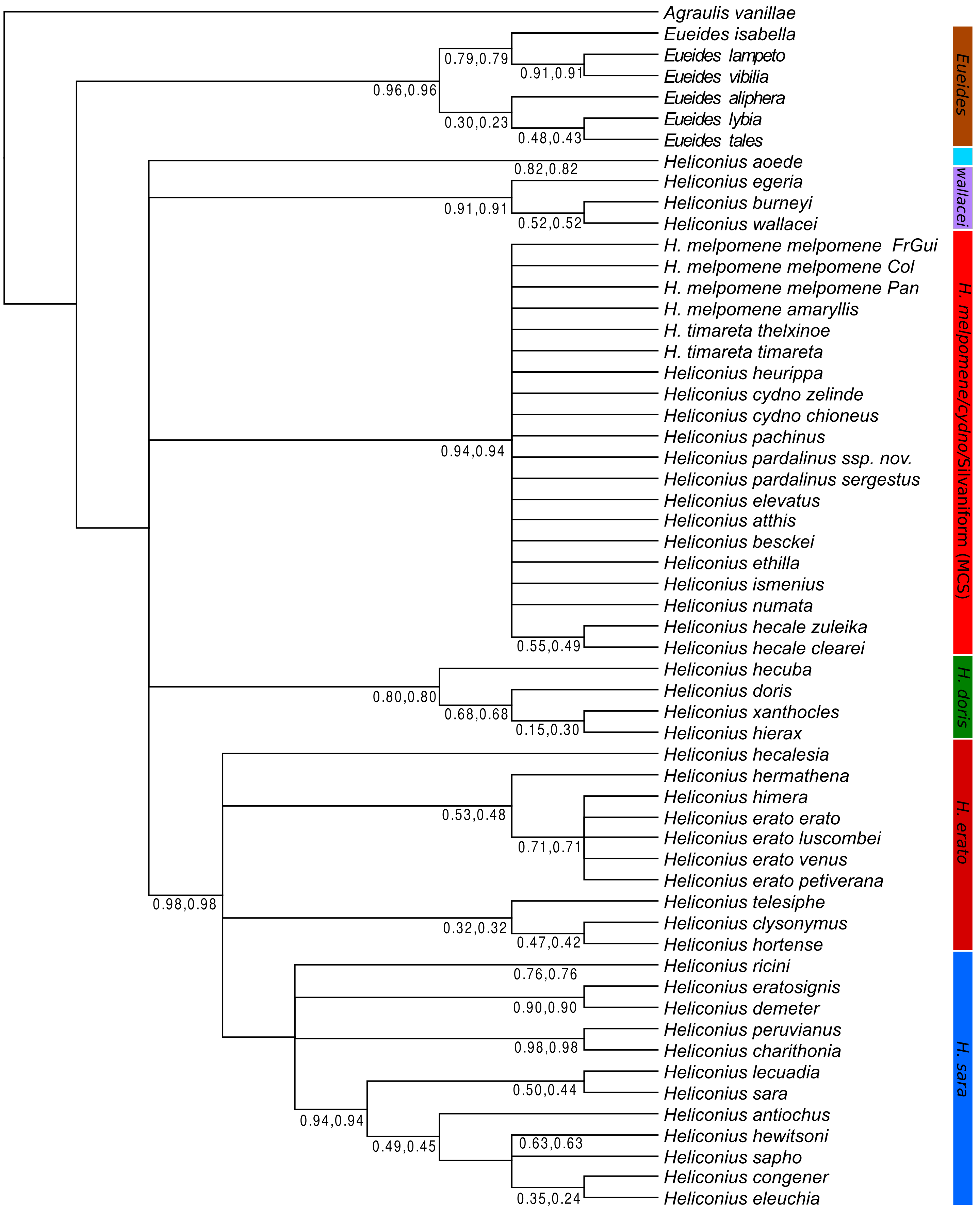


**Supplementary Figure 4. Autosomal gene trees disagree at most nodes.** 50% Majority Rule Consensus tree based on 6724 Agraulis-rooted gene trees. Branch labels indicate the Internode Certainty and Internode Certainty All support (Salichos et al. 2014). An IC=0.0 means that a given node has an equally frequent alternative in the distribution of gene trees, whereas IC=1.0 means that all trees contains this node. FrGui: French Guiana; Col: Colombia; Pan: Panama.

______________________________________________________________________


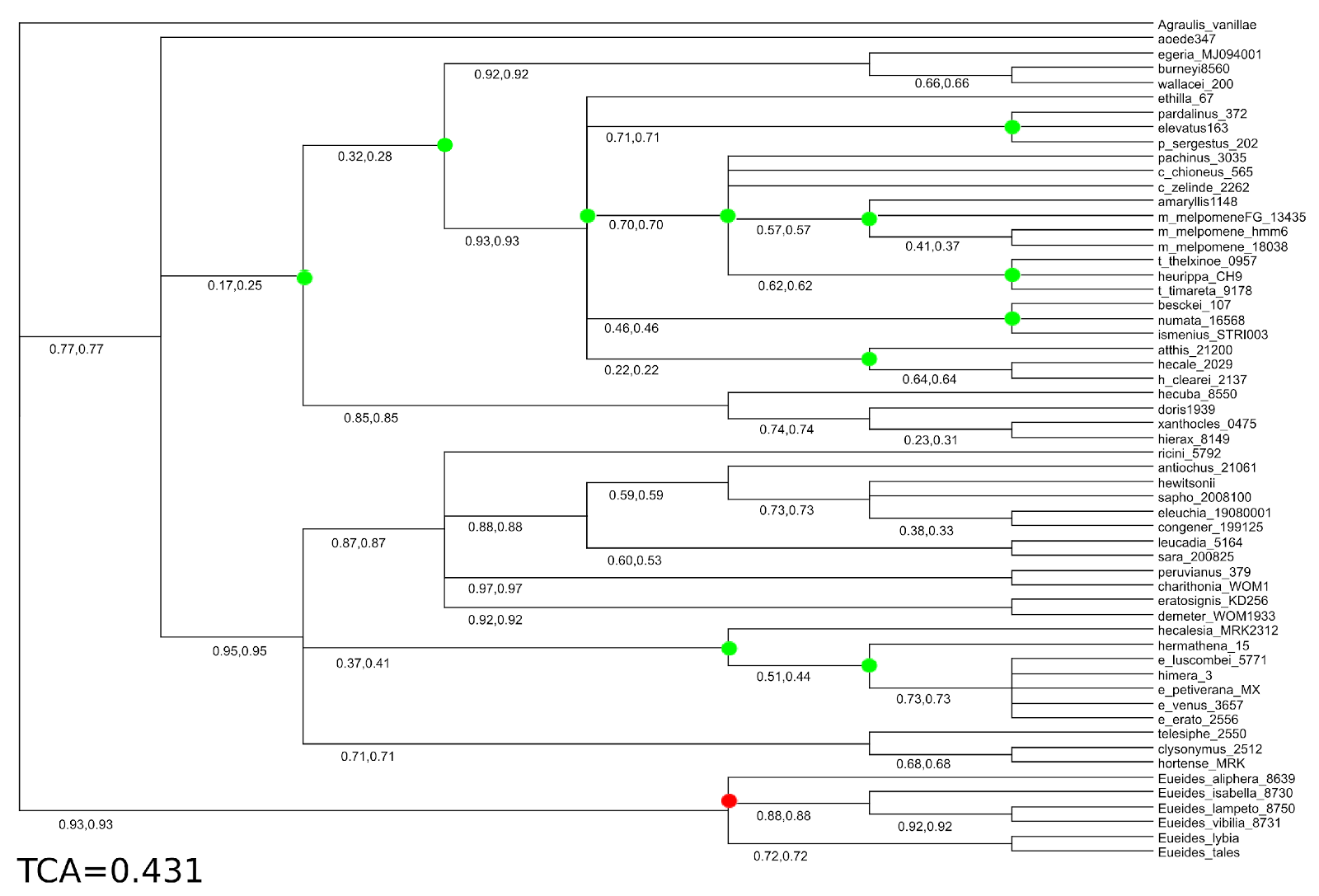


**Supplementary Figure 5. Higher congruence between gene trees results in more resolved consensus and higher Tree Certainty at the Z chromosome than at the autosomes.** 50% Majority Rule Consensus the Z-linked gene trees with IC/ICA support values indicated. Green dots indicate nodes unresolved in the autosomal 50% MRC, red dots nodes conflicting with the autosomal tree.


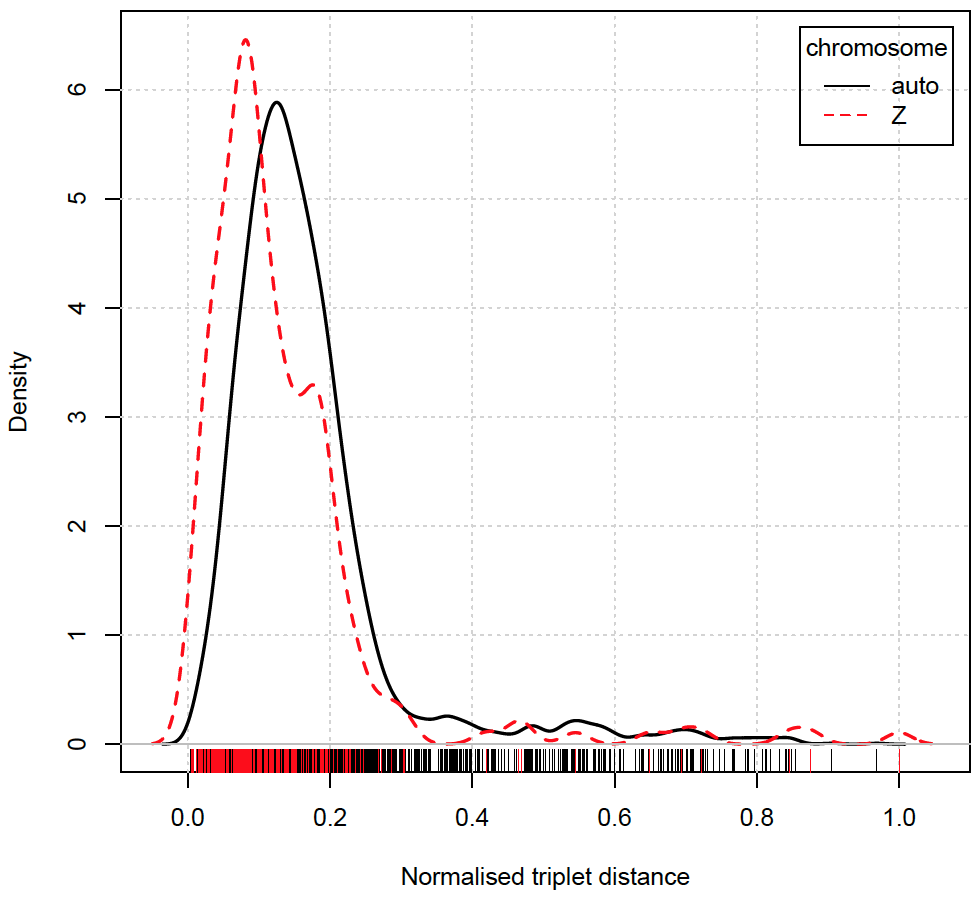


**Supplementary Figure 6. Autosomal and Z-linked genes are almost equally conflicted with the species tree (MP-EST).** For each gene tree we calculated the number of taxon triplets also found in the MP-EST estimate and normalized to a 0-1proportion. The smoothed distribution of normalized values is reported for autosomal and Z-linked gene trees.

**Supplementary Figure 7. ASTRAL-III MSC phylogeny for the Z (sex-linked) chromosome.** Numerical labels represent normalized quartet score, corresponding to the amount of incongruence around a branch.


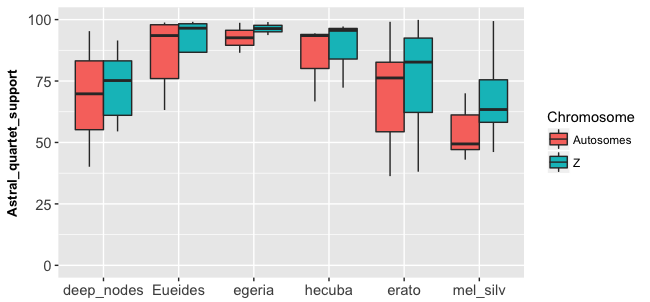


**Supplementary Figure 8. Gene tree incongruence is the highest in the *H. melpomene/*silvaniform clade.** The quartet support values from ASTRAL are lower when more gene trees do not contain a given quartet. Lower mean quartet support of all nodes in a given tree is indicative of greater incongruence.


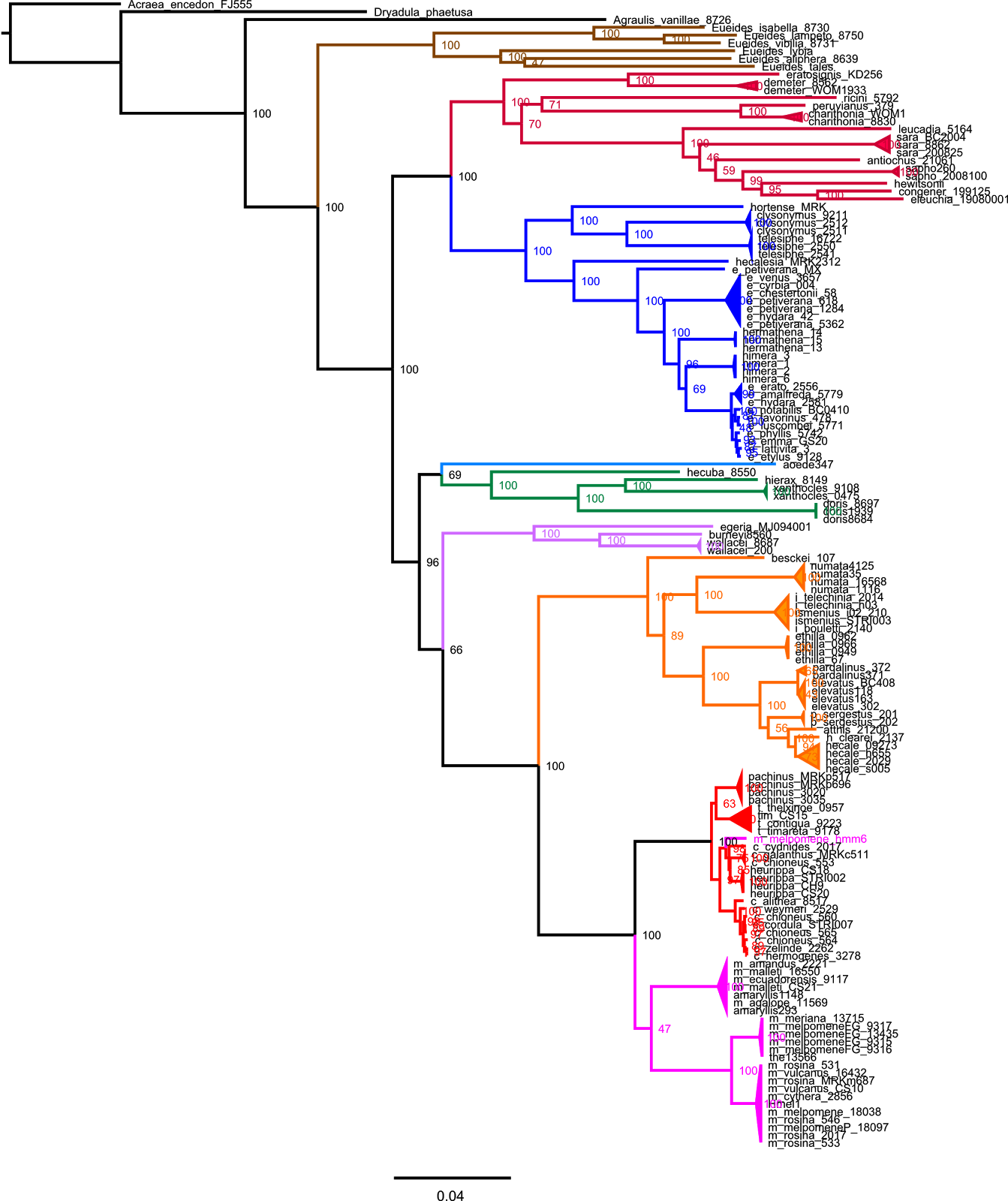


**Supplementary Figure 9. Whole-mitochondrial sequences “resolve” the radiation of *Heliconius*, although they conflict with autosomal and sex-linked signals.** A Maximum Likelihood tree (RAxML) with bootstrap support values. Colors indicate major clades. Scale bar in substitutions per site.


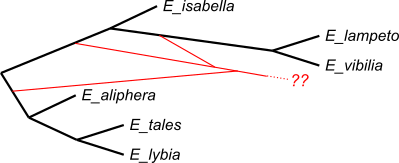


**Supplementary Figure 10**. **Inconsistent estimates of gene flow in autosomal gene tree-based MPL networks for *Eueides*, the sister genus of *Heliconius*, including 6/12 species.** The vertices of multiple branches may represent an artefactual “ghost lineage” corresponding to missing species.


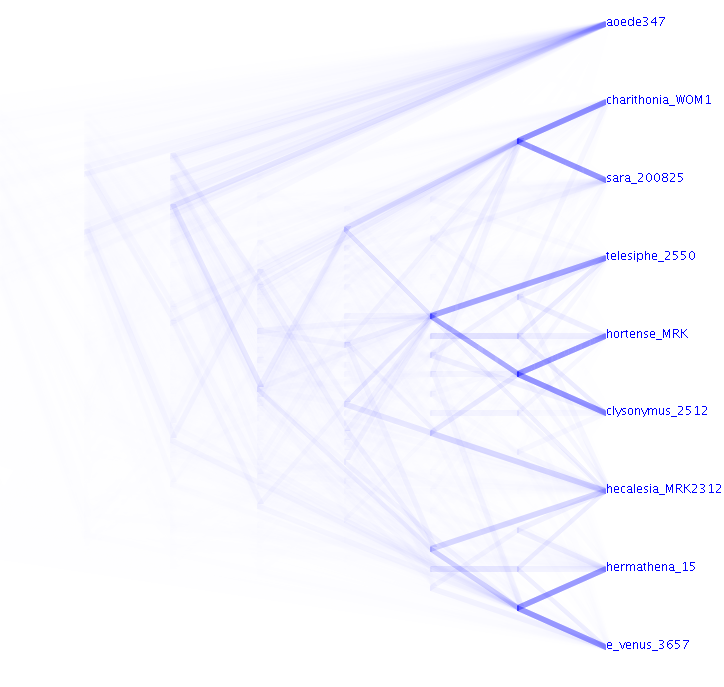


**Supplementary Figure 11. Placement of *H. hecalesia* is ambiguous across gene trees.** DensiTree plot of 6848 autosomal gene trees shows variable placement close to either *H. erato* or *H. telesiphe* clades.


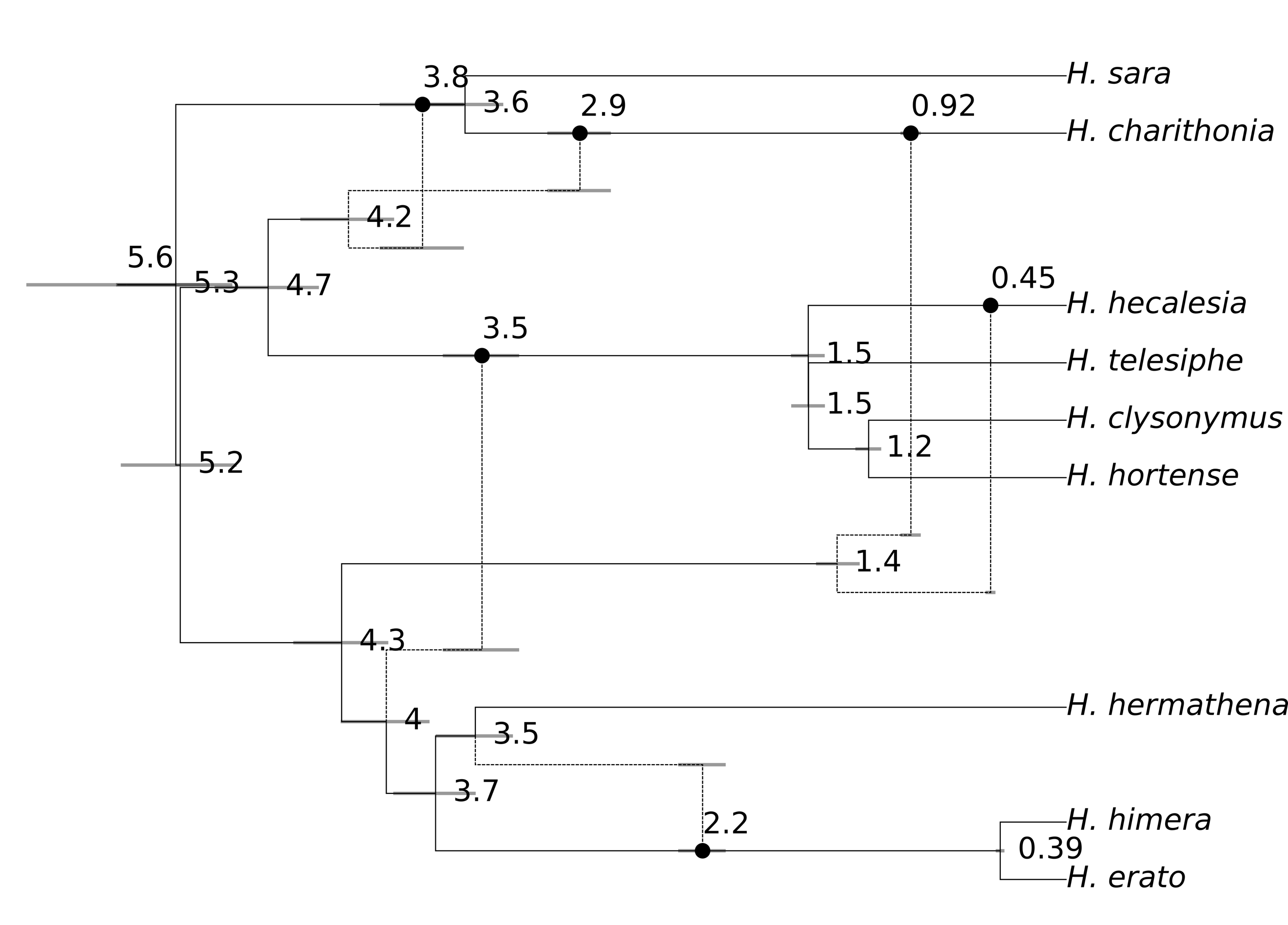


**Supplementary Figure 12. Multiple instances of introgression between species in the *SEC* clade are inferred at the *optix* locus responsible for red wing patterning (HE670865:310,000:460,000).** BEAST2 Bayesian species network inferred from 15 windows of 10kbp. Numbers indicate age of nodes in MY and horizontal bars represent the 95% highest posterior densities around age estimates. Vertical lines are admixture edges.


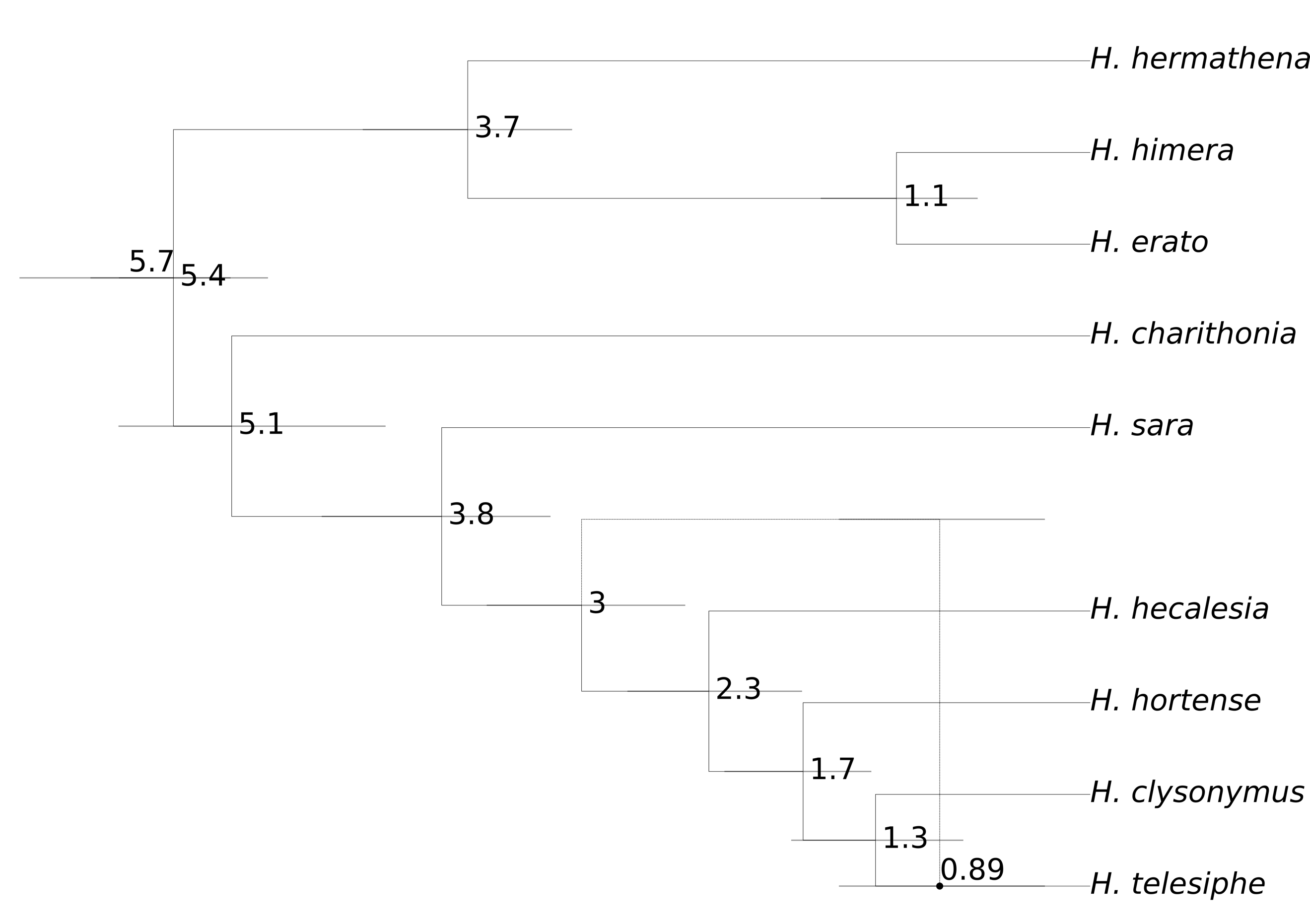


**Supplementary Figure 13. Bayesian model of introgression at the *cortex* locus (**HE667780**: 570-750 kbp) responsible for yellow wing patterns in the *SEC* clade.** BEAST2 coalescent species network inferred from 18 windows of 10kbp shows a topology different from the genome-wide species tree. Numbers indicate age of nodes in MY and horizontal bars represent the 95% highest posterior densities around age estimates. The vertical line is an admixture edge.


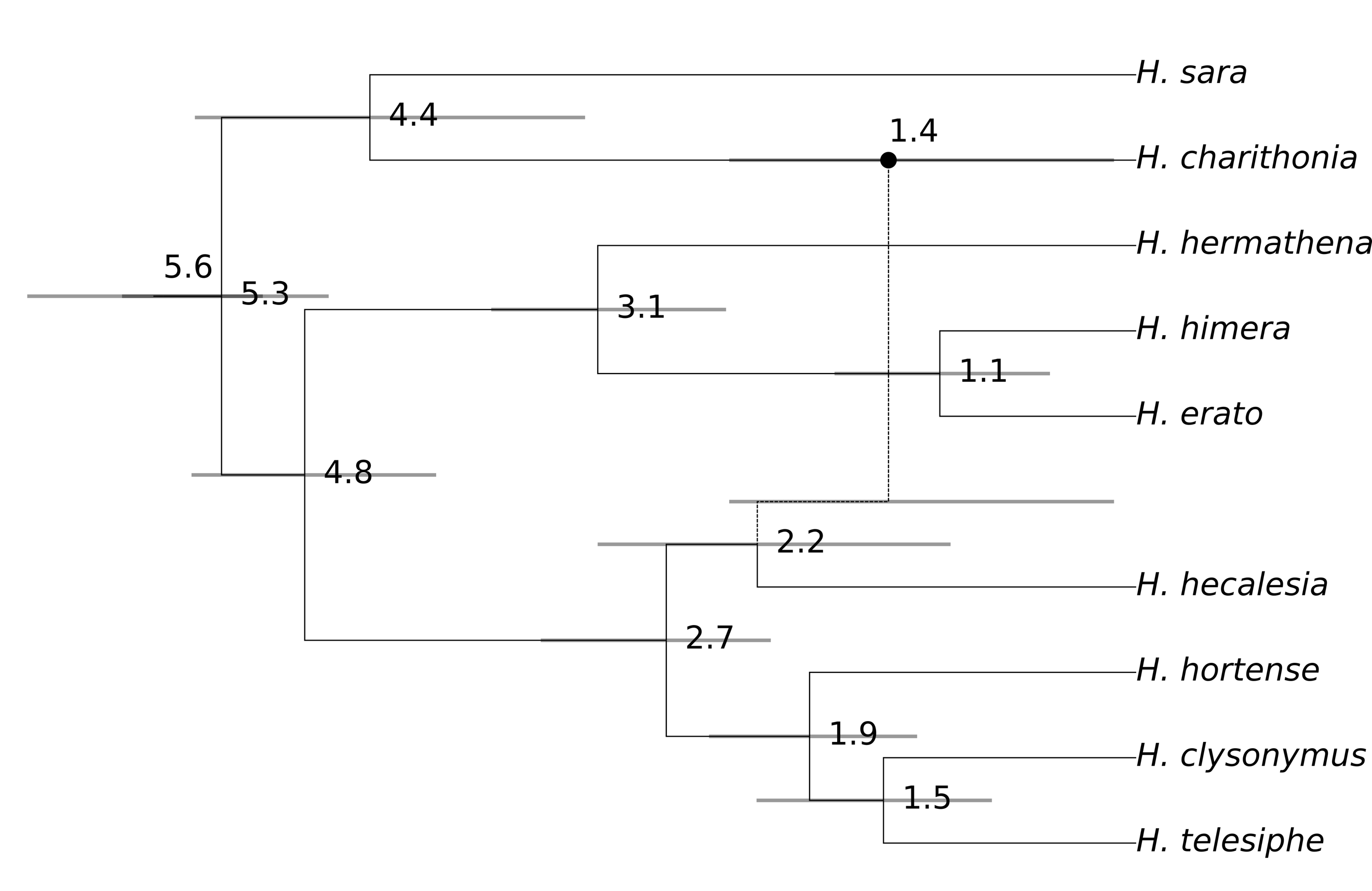


**Supplementary Figure 14. Bayesian model of introgression at the *Sd* locus (HE668478: 450-490 kbp) responsible for shape of wing patterns in the *SEC* clade.** BEAST2 network inferred from five windows of 10kbp. Numbers indicate age of nodes in MY and horizontal bars represent the 95% highest posterior densities around age estimates. The vertical lines is an admixture edge.
